# Declining Amazon biomass due to deforestation and subsequent degradation losses exceeding gains

**DOI:** 10.1111/gcb.16513

**Published:** 2022-11-23

**Authors:** Dominic Fawcett, Stephen Sitch, Philippe Ciais, Jean Pierre Wigneron, Celso H. L. Silva‐Junior, Viola Heinrich, Christelle Vancutsem, Frédéric Achard, Ana Bastos, Hui Yang, Xiaojun Li, Clément Albergel, Pierre Friedlingstein, Luiz E. O. C. Aragão

**Affiliations:** ^1^ Department of Geography, Faculty of Environment, Science and Economy University of Exeter Exeter UK; ^2^ Laboratoire des Sciences du Climat et de l'Environnement LSCE CEA CNRS UVSQ, Centre d'Etudes Orme de Merisiers Gif‐sur‐Yvette France; ^3^ INRAE, UMR ISPA Université de Bordeaux Villenave d'Ornon France; ^4^ Institute of Environment and Sustainability University of California Los Angeles California USA; ^5^ Jet Propulsion Laboratory California Institute of Technology Pasadena California USA; ^6^ Programa de Pós‐graduação em Biodiversidade e Conservação Universidade Federal do Maranhão São Luís Brazil; ^7^ School of Geographical Sciences University of Bristol Bristol UK; ^8^ FINCONs Group Milan Italy; ^9^ Center for International Forestry Research (CIFOR) Bogor Indonesia; ^10^ European Commission Joint Research Centre Ispra Italy; ^11^ Department of Biogeochemical Integration Max Planck Institute for Biogeochemistry Jena Germany; ^12^ European Space Agency Climate Office ECSAT, Harwell Campus Didcot Oxfordshire UK; ^13^ Mathematics and Statistics, Faculty of Environment, Science and Economy University of Exeter Exeter UK; ^14^ LMD/IPSL, ENS PSL Université, Ècole Polytechnique, Institut Polytechnique de Paris Sorbonne Université, CNRS Paris France; ^15^ Tropical Ecosystems and Environmental Sciences Laboratory São José dos Campos Brazil; ^16^ Earth Observation and Geoinformatics Division National Institute for Space Research São José dos Campos Brazil

**Keywords:** Amazon, biomass, carbon, deforestation, degradation, forest, growth, VOD

## Abstract

In the Amazon, deforestation and climate change lead to increased vulnerability to forest degradation, threatening its existing carbon stocks and its capacity as a carbon sink. We use satellite L‐Band Vegetation Optical Depth (L‐VOD) data that provide an integrated (top‐down) estimate of biomass carbon to track changes over 2011–2019. Because the spatial resolution of L‐VOD is coarse (0.25°), it allows limited attribution of the observed changes. We therefore combined high‐resolution annual maps of forest cover and disturbances with biomass maps to model carbon losses (bottom‐up) from deforestation and degradation, and gains from regrowing secondary forests. We show an increase of deforestation and associated degradation losses since 2012 which greatly outweigh secondary forest gains. Degradation accounted for 40% of gross losses. After an increase in 2011, old‐growth forests show a net loss of above‐ground carbon between 2012 and 2019. The sum of component carbon fluxes in our model is consistent with the total biomass change from L‐VOD of 1.3 Pg C over 2012‐2019. Across nine Amazon countries, we found that while Brazil contains the majority of biomass stocks (64%), its losses from disturbances were disproportionately high (79% of gross losses). Our multi‐source analysis provides a pessimistic assessment of the Amazon carbon balance and highlights the urgent need to stop the recent rise of deforestation and degradation, particularly in the Brazilian Amazon.

## Graphical abstract

We used satellite L‐Band Vegetation Optical Depth (L‐VOD) data to track Amazon biomass changes over 2011–2019 and sought to attribute these changes by modelling individual processes. We show that the Amazon above‐ground carbon has declined since 2012 as losses from deforestation and subsequent degradation outweigh gains from secondary forest regrowth and variable old‐growth forest changes.

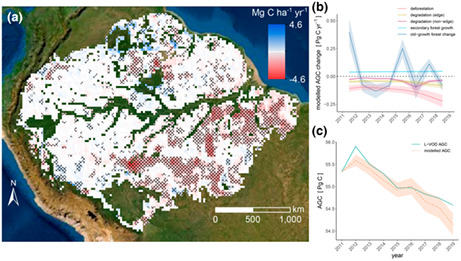

## 1 INTRODUCTION

The Amazon contains roughly a quarter of the world's above‐ground biomass carbon (AGC) stocks (Liu et al., 2015), but these are at risk from continuous deforestation, degradation and climate change (Doughty et al., 2015; Silva Junior et al., 2019). Deforestation can further contribute to regional drying, exacerbating climatic pressures (Staal et al., 2020). The Amazon old‐growth forest sink measured by forest inventories is decreasing (Brienen et al., 2015; Hubau et al., 2020), and forests in the southeastern Amazon are already acting as a net carbon source (Gatti et al., 2021) as a consequence of widespread forest degradation resulting from human and climatic pressures (Matricardi et al., 2020). Degradation is the partial loss of forest function and structural integrity from disturbance such as fire, selective logging and forest fragmentation which encompass the cascading effects of deforestation (Assis et al., 2022; Bullock et al., 2020; Matricardi et al., 2020; Qin et al., 2021; Silva Junior, Aragão, et al., 2020). Yet, degradation is not explicitly reported in national inventories and is not considered in commitments to reductions of greenhouse gas emissions (Silva Junior et al., 2021). Meanwhile, there are large areas of fast re‐growing secondary forest from post‐agricultural abandonment and fires in the Amazon. These forests have the potential to become a significant carbon sink, but they are subject to repeated disturbances (Heinrich et al., 2021; Wang et al., 2020; Yang et al., 2020). Understanding these opposite drivers of carbon dynamics is essential to forecast the future trajectory of the Amazon forest as a carbon sink and long‐term reservoir.

Satellite observations can reveal the locations and extent of processes causing uptake or release of carbon. High spatial resolution (30 m) optical data can map annual changes in forest cover (Hansen et al., 2013; Instituto Nacional de Pesquisas Espaciais [INPE], 2021; MapBiomas, 2021; Vancutsem et al., 2021) and disturbance events (Bullock et al., 2020; Vancutsem et al., 2021). In combination with static AGC maps (Baccini et al., 2012; Saatchi et al., 2011; Santoro & Cartus, 2021), these data can be used to estimate spatially specific immediate carbon emissions from deforestation and degradation (Harris et al., 2021). Yet, carbon gains or losses in intact or weakly disturbed forests are elusive.

Vegetation Optical Depth in the L‐band (L‐VOD) derived from the Soil Moisture and Ocean Salinity (SMOS) satellite passive microwave observations can be related to AGC at annual timescales (Brandt et al., 2018; Fan et al., 2019; Qin et al., 2021), which makes it a unique tool to map global carbon dynamics. L‐VOD can overcome limitations of optical sensors in tropical regions, since it is unaffected by clouds and remains sensitive to changes in high biomass values (Rodríguez‐Fernández et al., 2018). However, L‐VOD observations have a spatial resolution of 0.25° (~25 km). This is too coarse to attribute observed changes, since one grid‐cell can contain losses along with gains from old‐growth or secondary forest (Qin et al., 2021). So far, no attempts have been made to compare L‐VOD based ‘top down’ AGC changes to models representing individual processes leading to carbon gain or loss, such as deforestation, degradation, secondary forest growth processes and environmental factors affecting carbon changes of ‘intact’ old‐growth forests such as natural disturbances, CO_2_ fertilization and climate‐induced mortality.

Here we tackle this challenge by modelling estimates of inter‐annual changes in AGC associated with the processes of deforestation, degradation and secondary forest growth for the entire Amazon region. This model is integrated at high spatial resolution thanks to new datasets such as the biomass maps from ESA CCI (Santoro & Cartus, 2021) (100 m) and multi‐temporal land‐cover classification datasets (MapBiomas, 2021; Vancutsem et al., 2021) (30 m). Combined with L‐VOD‐based AGC changes to track carbon in old‐growth forests, this model allows a timely assessment of Amazon carbon dynamics. Results of this work provide new data to assess national carbon budgets, define baselines for land‐based mitigation efforts and evaluate models used for future projections (Ciais et al., 2020).

## 2 MATERIALS AND METHODS

### 2.1 Satellite data processing

#### 2.1.1 AGC product retrieved from L‐VOD

The L‐VOD AGC data were derived from the SMOS passive microwave satellite images L‐VOD product (version 2.0) developed using the SMOS‐IC algorithm (Fernandez‐Moran et al., 2017; Wigneron et al., 2021). This SMOS‐IC V2 L‐VOD product consists of global data with 1–3 day revisit times from ascending (ASC) and descending (DESC) orbit acquisitions.

Extensive filtering of the L‐VOD data is necessary due to the influences of land‐cover and radio frequency interference (RFI) on the L‐VOD retrieval. Pixels affected by water and urban areas as well as steep terrain were excluded based on quality flags from auxiliary datasets (Brandt et al., 2018). L‐VOD retrieved from passive microwave observations can also be influenced by sub‐canopy flooding, amplifying the intra‐annual variations in L‐VOD independent of biomass and vegetation water content changes (Bousquet et al., 2021). When comparing annual values derived only from the wet season for most of the Amazon, we expect a reduced impact of this effect on biomass change estimates and trends, however absolute values in inundated areas may be underestimated. Therefore, in addition to SMOS‐IC data flags, a classification which can better distinguish inundated forest areas derived from synthetic aperture radar data (Hess et al., 2015) was used over the Amazon river basin to mask L‐VOD grid‐cells which are more than 25% inundated at high water levels. This threshold is less conservative than the 10% open water threshold used for SMOS‐IC data flags (Fernandez‐Moran et al., 2017) to avoid masking vast expanses of seasonally partially flooded forests.

Observations with a brightness temperature deviation from modelled values (TB‐RMSE) of greater than 8 K were excluded as they indicate high RFI (Brandt et al., 2018; Fan et al., 2019). Both ASC and DESC data were considered to ensure a greater data record. While Qin et al (Qin et al., 2021) only used ASC data to study the Brazilian Amazon, the inclusion of DESC data was deemed appropriate due to the enhanced filtering procedure (Thoning et al., 1989) used. For each trimester, we investigated the difference between ASC and DESC mean values and if it exceeded 0.05 discarded the respective data if TB‐RMSE >5 K. Daily values are then combined and if ASC and DESC are available on the same day, the value with lower associated TB‐RMSE was used. Outliers beyond 2 standard deviations from the trimester mean values were also excluded (Brandt et al., 2018).

Following this pre‐processing, a curve fitting method was applied to the remaining daily data to extract a smoothed time series and the trend curve with seasonality removed (see Supplementary Materials for further details). Three different methods for deriving annual L‐VOD aggregate index values were applied: (1) Maximum of the smoothed curve fit, (2) Mean of the smoothed curve fit and (3) Mean of the trend curve. These values were derived over a 4‐month window from January to April of the respective year as these have high Amazon basin averaged precipitation and soil moisture storage, particularly in the southern Amazon (Li et al., 2006; Liang et al., 2020). This approach was selected over aggregating values for an entire calendar year as it should further decrease sensitivity to inter‐annual plant water content variations and increase the potential of the L‐VOD index to reveal year‐to‐year differences associated with deforestation. For Brazil deforestation peaks in May, on average 3 months after the rainy season, and leads the peak of fire in August to September (Aragão et al., 2008).

The mean of AGC estimates derived from these three indices was used for the analysis and the standard deviation between estimates included in the uncertainty.

The ESA CCI biomass (v2) map with reference year 2017 (Santoro & Cartus, 2021) was used to calibrate the annual L‐VOD index values to AGC. The ESA CCI product was favoured over other datasets with reference year 2010 (Avitabile et al., 2016; Baccini et al., 2012; Saatchi et al., 2011) due to a complete L‐VOD data record for the full wet season of the reference year and the previous use of ESA CCI in derivation of secondary forest growth curves used in this study (Heinrich et al., 2021). We fitted a four parameter function curve (Fan et al., 2019) to the L‐VOD indices and corresponding ESA CCI biomass values in 2017:
(1)

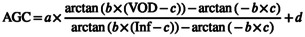

The corresponding values of *a–d* for each product and their standard errors can be found in Table S1, Inf was set to 10^10^.

Despite extensive filtering, some regional anomalies were evident in year‐to‐year AGC changes derived from L‐VOD (see Supplementary Materials). These cells were excluded from the analysis by masking cells showing greater than 20 Mg C ha^−1^ increase in any one year. This is a permissive threshold, considerably higher than the potential forest carbon accumulation rate for the biome (up to 6 Mg C ha^−1^ year^−1^; Cook‐Patton et al., 2020), in order to remove the most anomalous cells.

#### 2.1.2 Land‐cover datasets

The Mapbiomas Amazonia ‘Collection 2’ (MapBiomas, 2021) product represents an annual land‐cover classification derived from Landsat data using the random forest algorithm. Collection 2 covers the entirety of the Amazon biome from 1985 to 2018. Deforested areas were identified where the Mapbiomas classification changed from forest in the previous year to pasture, agriculture or vegetation‐free land‐cover.

Annual secondary forest extent was derived from a reclassification of Mapbiomas C2 (Silva Junior, Heinrich, et al., 2020) for pixels that were not forest for at least 1 year prior to transitioning to forest class. The secondary forest age was calculated as successive years where a pixel remains forest while forests regrowing since before 1985 could not be identified by this dataset.

Areas of forest degradation were extracted from the tropical moist forest (TMF) land cover change dataset (Vancutsem et al., 2021). The TMF dataset defines degradation as a disturbance in the tree canopy cover that is visible from space over a short period of time (less than 2.5 years) leading to a loss of biodiversity and/or carbon storage. This includes events such as logging, fire, windthrow and drought induced tree mortality. The degraded pixels remain forested. Baselines of degraded forest considering degradation events throughout the whole time‐series of available data (1990–2018) were generated to differentiate degraded from old‐growth forest and new degradation events identified for each year were used to derive degradation losses in 2011–2018.

Where deforestation occurs, the forest edges at the deforestation front are subject to selective logging and fires. These processes are widespread but not always identified by optical data and we therefore modelled forest edge degradation explicitly. These processes have been found to affect forest areas up to 120 m from the deforested edge (Silva Junior, Aragão, et al., 2020). An annual 120 m buffer‐based classification of forest edge areas was used as a reference and these areas were excluded from the disturbance dataset introduced above.

An old‐growth forest layer was derived for each year by excluding previously degraded (since 1990) and secondary forest areas from the Mapbiomas forest classification.

Changes between land‐cover types and comparisons to ensure consistency between datasets (such as forest extents between Mapbiomas and TMF map) were performed at the original 30 m spatial resolution of the data, prior to any resampling to fractional covers for computations with coarser resolution datasets.

### 2.2 Biomass change modelling

To model biomass change on the basis of the land‐cover classification we applied a number of simplified methodologies described in the following. A flowchart overview of the datasets involved is provided in Figure S1. Where AGC change relied on reference data derived from the ESA CCI 2017 biomass product, the change was estimated at 1 km resolution, otherwise at the original resolution of the datasets. Outputs were then aggregated to 0.25° to match L‐VOD grid‐cells and combined with old‐growth forest change to represent total AGC change per year. All biomass changes associated with conversion between forest classes or non‐forest are assumed to occur within the year that they are detected.

To estimate a biomass loss factor associated with non‐edge degradation, reference AGC values for old‐growth forest and degraded forest were extracted from the ESA CCI biomass product at 100 m spatial resolution using the land‐cover datasets and yielded median old‐growth AGC values of 126.4 ± 27.3 Mg C ha^−1^ and median degraded AGC values of 81.78 ± 27.1 Mg C ha^−1^. Based on these values we estimate a 35.3% reduction of AGC associated with disturbance events that result in further degraded forest for non‐edge degradation. This value is greater than AGC reduction associated with logging but smaller than that associated with fire from LiDAR datasets; however, it was previously found that disagreements between pan‐Amazonian biomass maps and LiDAR data were high and these maps generally had smaller differences between old‐growth and burnt forest AGC (Longo et al., 2016).

To obtain regional reference values of old‐growth forest for mixed grid cells, we applied a circular filter with 1° radius to calculate median values from the biomass map masked by old‐growth forest areas (cells with >90% old‐growth forest cover). The same spatial filtering method was applied to obtain reference values of changes in old‐growth forest AGC from L‐VOD; however, this required a two‐step filtering approach with 2.5, then 5° radius to provide a reference value for every grid‐cell due to fewer >90% old‐growth grid‐cells at 0.25° resolution. The change in old‐growth forest AGC for >90% old‐growth grid cells was calculated as the residual of total L‐VOD AGC change and the modelled changes of the different processes as shown in Equation (1). In these reference cells, the combination of changes attributed to the different processes is therefore equal to the L‐VOD AGC change.

(2) $$\Delta {\mathrm{AGC}}_{\mathrm{old}-\text{growth}\_\mathrm{ref}}=\Delta {\mathrm{AGC}}_{L-\mathrm{VOD}}-\left(\Delta {\mathrm{AGC}}_{\text{deforestation}}+\Delta {\mathrm{AGC}}_{\text{degradation}}+\Delta {\mathrm{AGC}}_{{\mathrm{SF}}_{\text{growth}}}\right).$$

For secondary forest and forest edges, we used growth and loss curves respectively to describe the AGC (change) per year as a function of the secondary forest and edge ages. We used a secondary forest growth curve that was derived for the north‐west Brazilian Amazon (see Table S2) (Heinrich et al., 2021) where climatic conditions are expected to be most representative of the entirety of the Amazon biome and the estimated growth rates are similar to a pan‐tropical growth curve (Wang et al., 2020). Previous pan‐Amazonia scale studies have used baseline accumulation rates for young and old secondary forests (Smith et al., 2021). The change in secondary forest AGC is derived from the growth curve using secondary forest age and accounting for changes in secondary forest extent.

To quantify biomass loss in forest edges, we apply a loss model as a function of edge age, derived from airborne LiDAR observations (Table S2) (Silva Junior, Aragão, et al., 2020).

Deforestation of old‐growth forest, degraded forest and secondary forest was distinguished. The biomass lost through deforestation per year was calculated as the fraction of deforested area multiplied by old‐growth forest reference AGC or a degraded reference AGC after applying the forest edges loss model. The secondary forest AGC deforested was calculated using the growth function (Table S2) and age per deforested secondary forest pixel.

### 2.3 Statistical analysis

Trends in L‐VOD derived and modelled AGC at the 0.25° grid‐cell level were robustly fit using the Theil‐Sen trend estimator and their significance estimated at the 95% confidence level using the ‘trend’ package (v. 1.1.4) in R (Pohlert, 2020).

To investigate the correspondence between L‐VOD derived and modelled AGC change we calculated Pearson's r and mean absolute deviation (MAD) both for trends in AGC for grid‐cells with less than 90% old‐growth forest fraction in 2018.

To generate a total uncertainty estimate for modelled AGC change we combined the uncertainties associated with the different processes and the annual changes using root sum of squares. Results from an alternative method using summation of annual uncertainties are reported in the SI.

## 3 RESULTS

### 3.1 Trends in Amazon carbon stocks

**FIGURE 1:**
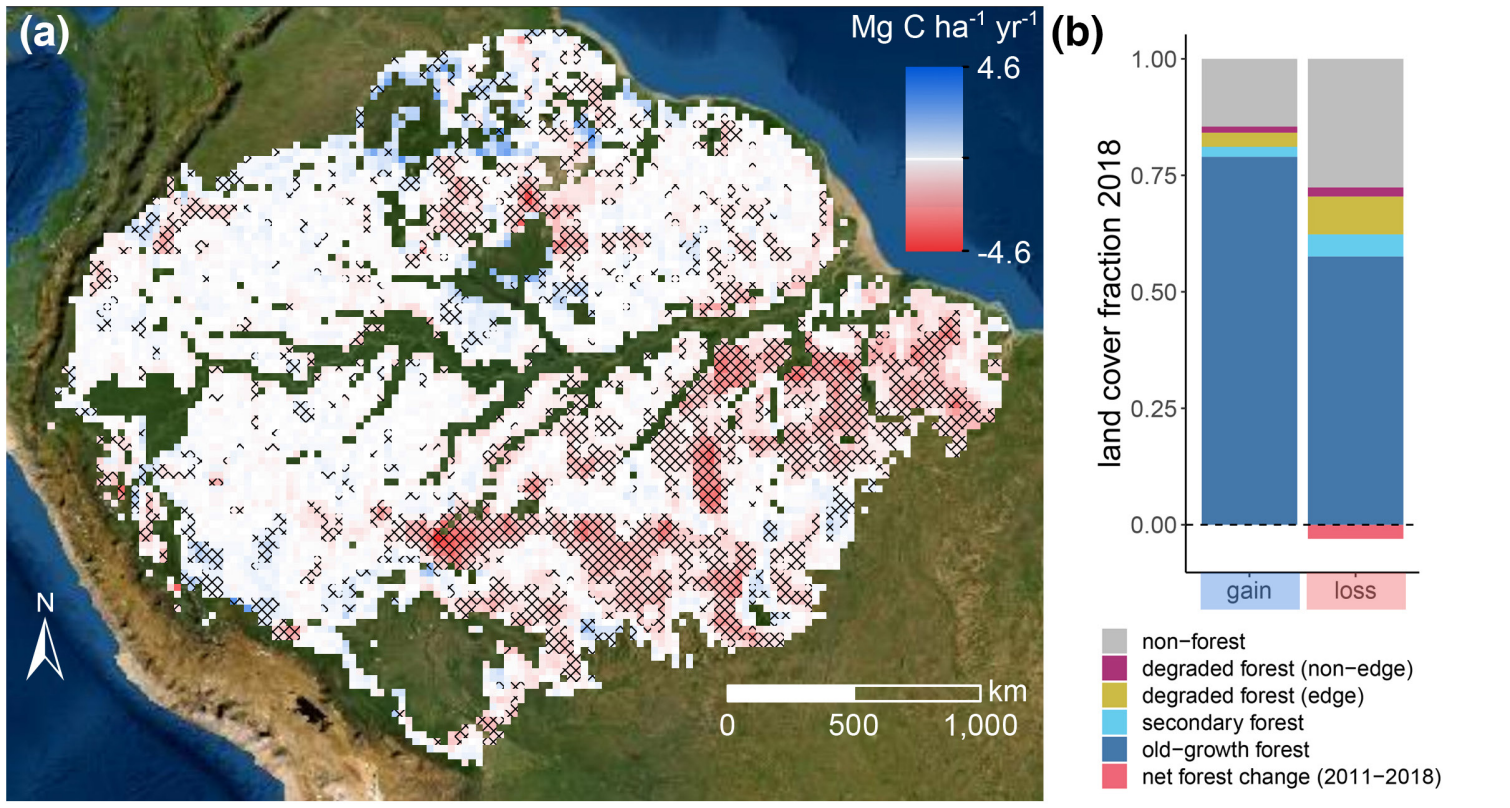
Trends in AGC and the associated forest cover fractions. (a) AGC trends (2011–2019) over the Amazon biome. Grid‐cells where trends are significant are indicated with cross‐hatches and cells where reliable data were not available are omitted (e.g. flooded areas and regional anomalies). (b) Mean land cover fractions in 2018 averaged over grid‐cells showing significant positive (gain) and negative (loss) trends. Displayed are the proportions of old‐growth, degraded (edge and non‐edge), secondary forest and non‐forest area. Edge degradation includes forest within 120 m distance from human‐made forest edges (Silva Junior, Aragão, et al., 2020). The net change in forest area fraction relative to 2011 is also indicated (~0 for gain cells, −0.03 or 4% reduction for loss cells). Basemap sources: Esri, DigitalGlobe, GeoEye, Earthstar Geographics, CNES/Airbus DS, USDA, USGS, AeroGRID, IGN, and the GIS User Community.

We used L‐VOD data to quantify changes of AGC within 0.25° grid‐cells over the entire Amazon biome. Trends in AGC for the 2011–2019 period reveal areas that were losing or gaining carbon over the last decade (Figure 1a). Areas with significant carbon losses were ~5 times greater than areas showing significant gains. Carbon gains occurred in areas that are mostly covered by old‐growth forest, here defined as forests that were not detected as disturbed since the beginning of the observation period (degradation mapping since 1990; Vancutsem et al., 2021). On average, gains were not associated with net increase in forest cover (Figure 1b). Contiguous areas of increasing AGC are found in the South‐West Amazon (Figure 1a).

Losses correspond spatially with the arc of deforestation and occur predominantly in South‐Eastern Amazon with rates up to ~4.6 Mg C ha year^−1^. The forest cover classification reveals that grid‐cells with significant losses experienced a 4% mean decrease in forest area. Further, 21% of the remaining forest area in these loss regions is classified as disturbed, including extensive degraded areas as a consequence of fires, logging and fragmentation (Matricardi et al., 2020) but also areas of regrowing secondary forest after agricultural abandonment (Figure 1b).

### 3.2 Spatial distribution of AGC change drivers

**FIGURE 2:**
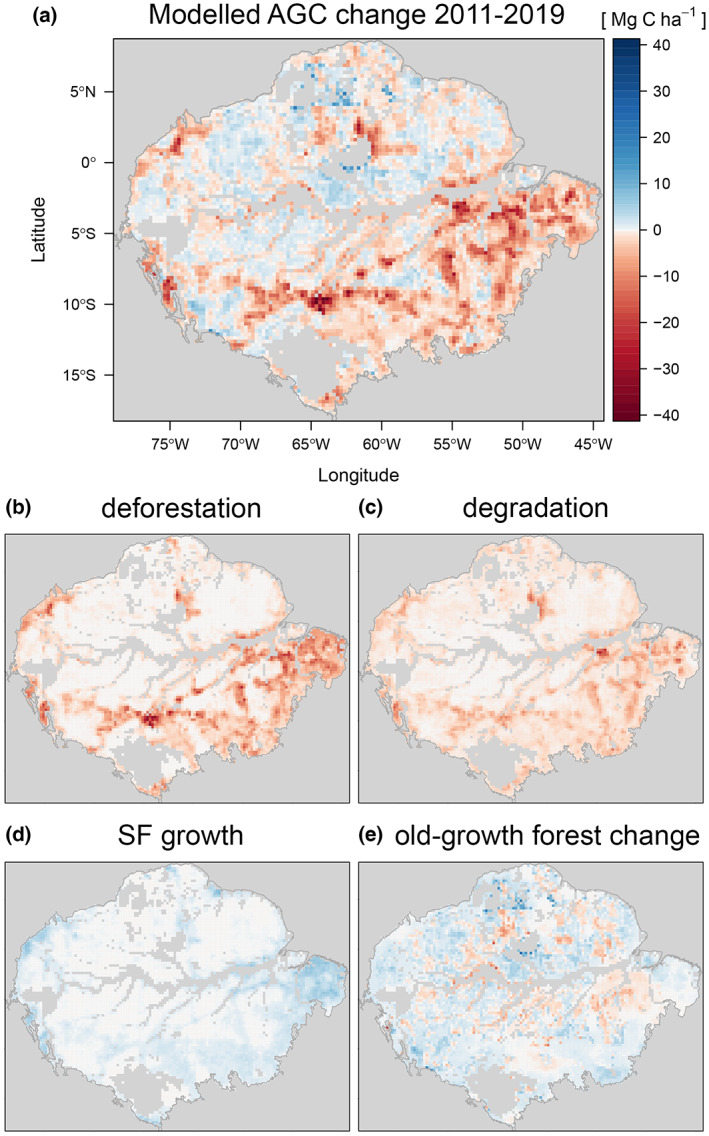
Total AGC changes associated with different processes. (a) Net change of AGC inferred by the combination of modelled and old‐growth forest changes over the 2011–2019 period, (b–d) modelled AGC change due to processes of (b) deforestation, (c) degradation, (d) secondary forest growth and (e) AGC change due to other processes within old‐growth forests inferred by combining old‐growth forest cover with the L‐VOD AGC change residual within proximal >90% old‐growth forest cover grid‐cells.

For each 0.25° grid‐cell, we modelled AGC change from deforestation, degradation and secondary forest growth separately by combining fine spatial resolution (30 m) land‐cover and change classification data with a static high resolution biomass map (MapBiomas, 2021; Santoro & Cartus, 2021; Vancutsem et al., 2021). Changes in old‐growth forest within mixed grid‐cells were inferred from the residual L‐VOD AGC changes of proximal grid‐cells with >90% old‐growth forest (see Methods). The combination of these estimates is further referred to as ‘modelled AGC change’ and reveal the spatially distinct drivers of AGC changes in the Amazon (Figure 2a) and the contribution of different loss and gain processes (Figure 2b–e).

Trends derived from modelled estimates from all the above processes over the 2011–2019 period showed moderate correspondence spatially with total L‐VOD AGC trends (*R* ^2^: .46, excluding grid‐cells with >90% old‐growth forest, Figure S2a), while agreeing on positive or negative trends for 89% of this area (Figure S3). There were similar correlations when considering only the most disturbed grid‐cells (Figure S2b). Investigating differences between trends in L‐VOD AGC and the modelled values reveals that relative errors are highest in areas with greater agricultural land‐cover fractions (Figure S4) and modelled AGC losses are greater in areas experiencing a small‐to‐moderate amount of deforestation and degradation, particularly in the Western Amazon (Figure 2, Figure S5).

While hotspots of deforestation activity are responsible for the largest local losses (Figure 2b), the associated degradation greatly increases the area and magnitude of biomass losses (Brinck et al., 2017; Matricardi et al., 2020). Forest degradation closely follows spatial patterns of deforestation due to fire and logging‐related biomass loss at forest edges (Silva Junior, Aragão, et al., 2020). We observe that degradation hotspots are concentrated around floodplains, particularly the Branco floodplain in North‐Western Brazil (Figure 2c), which have been described as an Achilles' heel of the Amazon due to fire vulnerability (Flores et al., 2017).

Secondary forest growth is concentrated in the fragmented landscapes of the eastern Brazilian Amazon (Heinrich et al., 2021) and along western fringes in Peru and Colombia (Figure 2d). The old‐growth forest changes inferred from L‐VOD (Figure 2e) indicate net AGC gains in the Western Amazon while regions of the South‐Eastern Amazon are a carbon source. We also find L‐VOD AGC trends in Amazon‐wide old‐growth forest areas are negatively correlated with temperature increase for both long‐term (1981–2018) and short‐term (2011–2018) temperature trends, mostly influenced by the South‐Eastern Amazon (Figures S6 and S7).

### 3.3 Process‐level AGC change over time

**FIGURE 3:**
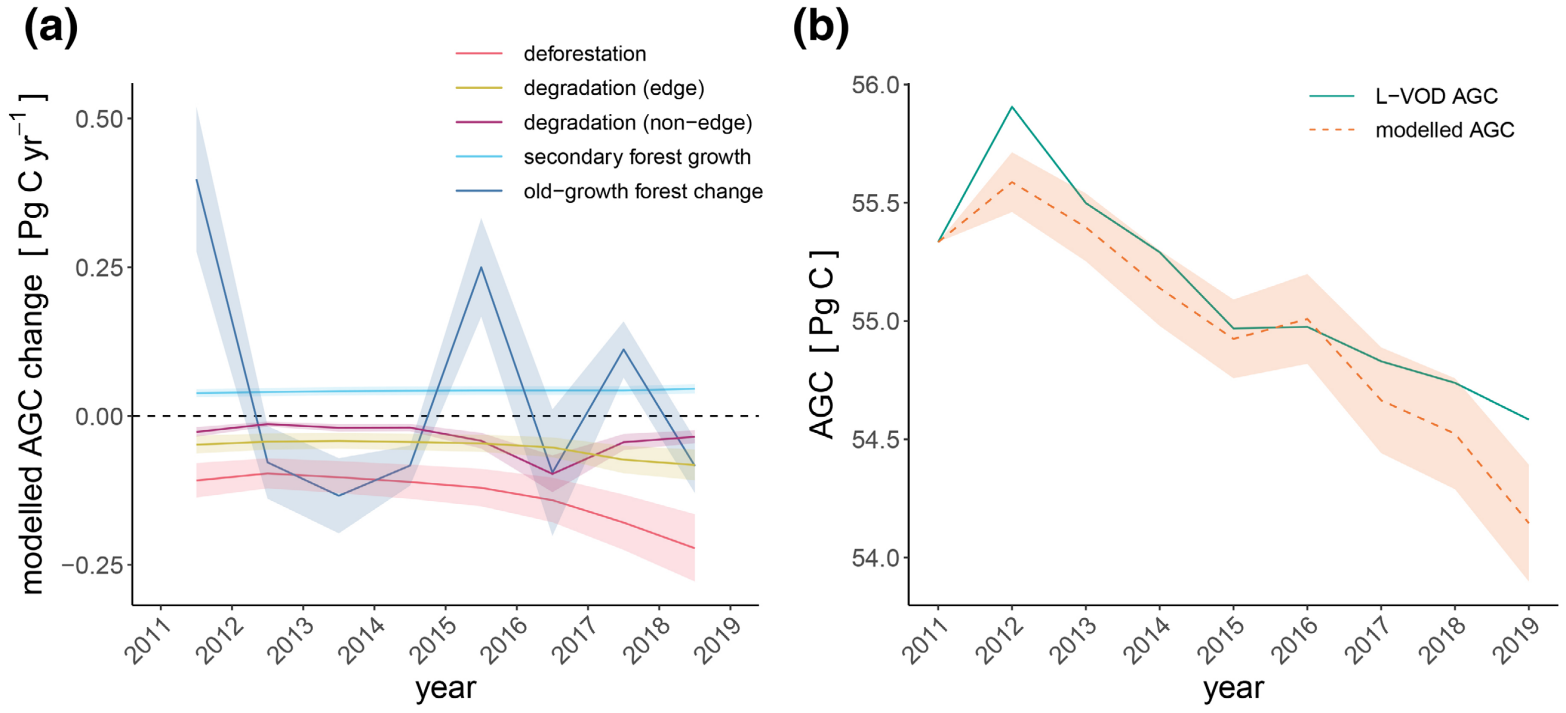
Time series of Amazon AGC changes. Time series showing (a) annual changes in AGC over the Amazon for 2011 up to 2018 as modelled for each process and inferred from L‐VOD (old‐growth forest change) and (b) total AGC from 2011 to 2019 over the Amazon, modelled and inferred from L‐VOD, where values represent AGC at the beginning of the respective year (January–April). Ribbons represent uncertainties associated with the ESA CCI biomass map (±1 SD, for deforestation, edge and non‐edge degradation loss), from the secondary forest growth model (±1 SD of average growth rate) and uncertainties reported for old‐growth forest change inferred from ±1 SD of the ESA CCI biomass map used for calibration of L‐VOD AGC and the three L‐VOD indices (see Section 2). The total modelled AGC in (b) includes the combination of these uncertainties while for L‐VOD AGC the ~30% error is omitted for the purpose of better visualization of the differences between years and dataset means.

L‐VOD reveals that AGC of the Amazon biome has decreased by 1.32 ± 0.4 Pg C between 2012 and 2019. The AGC decrease follows an increase of 0.57 ± 0.18 Pg C from 2011 to 2012 which is also evident in the old‐growth forest response (Figure 3a). After this initial increase, reductions in AGC within old‐growth forests over the following years suggest that these forests may have transitioned to a net source of carbon for the period 2012 to 2019 (−0.11 ± 0.18 Pg C), with high inter‐annual variability (Figure 3a).

Annual AGC changes modelled using our bottom‐up bookkeeping approach including old‐growth forest variations reproduce the decrease of Amazon carbon stocks inferred from the annual L‐VOD AGC values (Figure 3b). However, the modelled increase from 2011 to 2012 is smaller, indicating that part of the L‐VOD AGC additional increase lies in areas not dominated by old‐growth forests, including secondary forest, recovering degraded forest, pasture and croplands. Further, modelled losses since 2016 are greater than those inferred by L‐VOD AGC.

We find that deforestation accounted for a mean loss of 135 Tg C year^−1^ over the Amazon biome for the 2011–2019 period with an increase since 2012. As deforestation creates new forest edges, a similar increase in edge‐related carbon loss with a mean loss of 54 Tg C year^−1^ was identified. Non‐edge degradation has a distinct peak in 2016 with losses of 97 Tg C year^−1^.

Carbon gains by regrowing secondary forests saw a significant (*p* < .05) but modest increase from 39 Tg C year^−1^ in 2011 to 46 Tg C year^−1^ in 2018. However, we also observe a considerable increase in deforestation of secondary forest areas (Figures S8 and S9).

**FIGURE 4:**
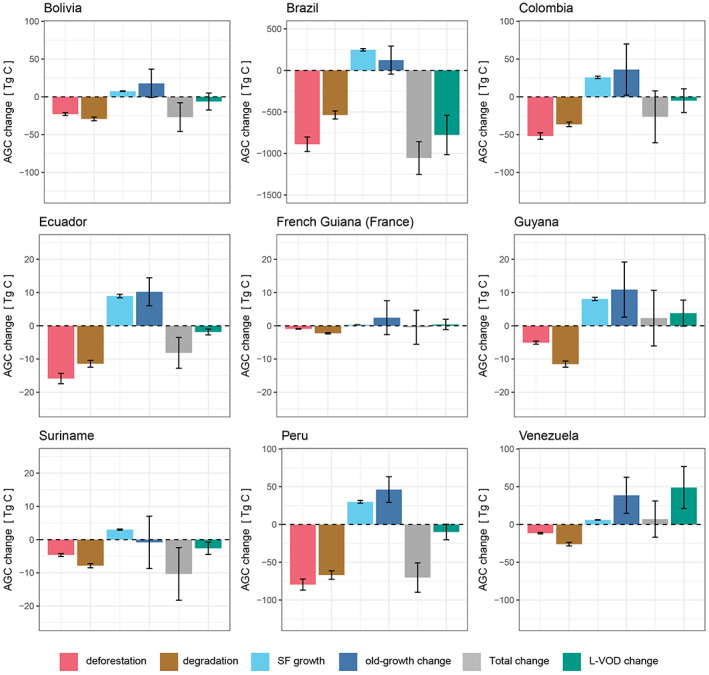
Country scale AGC changes in the Amazon forest from 2011 to 2019. AGC change associated with different processes (deforestation, degradation, secondary forest growth, old‐growth forest change), combined and L‐VOD inferred AGC change for parts of the Amazon forest divided by country, from the beginning of 2011 to the beginning of 2019. Note the different *y*‐axes to visualize changes for smaller countries. Whiskers represent uncertainties associated with the ESA CCI biomass map (±1 SD, for deforestation, edge and non‐edge degradation loss), from the secondary forest growth model (±1 SD of average growth rate) and uncertainties reported for old‐growth forest change and L‐VOD change inferred from ±1 SD of the ESA CCI biomass map used for calibration of L‐VOD AGC and the three L‐VOD indices (see methods). The total modelled changes include the combination of these uncertainties.

We also report AGC change per process in Amazonia of each of the nine countries intersecting the Amazon biome (Figure 4, Table S3) and their land‐cover fractions (Figure S10). The net modelled change in carbon stocks is negative for all countries, except Venezuela and Guyana. In Brazil, a net loss of 1.05 ± 0.2 Pg C is found since 2011 and it accounts for 78.7% of gross Amazon losses. In contrast, Brazil holds 63.5% of the total considered Amazon AGC stocks. Other large losses occur in Peru (−70.3 ± 19.4 Tg C [−1.2%]) and in Colombia (−26.5 ± 34.4 Tg C [−0.6%]). In most countries, deforestation losses are greater than degradation‐induced losses. For the Brazilian Amazon, degradation accounts for 37.6% of losses which is in line with previous estimates from inventories and bookkeeping models (Aguiar et al., 2016; Berenguer et al., 2014). For years following the large La Niña event in 2011, the old‐growth forests showed net AGC decreases for multiple countries including Brazil (Figure S11, Table S4).

Brazil's large AGC losses are confirmed by L‐VOD inferred AGC changes which show a 2.2% reduction in AGC since 2011. For other countries the trends in L‐VOD AGC mostly agree (Table S5), but there is low confidence in overall increase or decrease in AGC.

## 4 DISCUSSION

### 4.1 Spatial patterns of Amazon AGC changes

The largest losses of AGC in the Amazon occur along the arc of deforestation. Besides initial removal of biomass, deforestation‐induced forest fragmentation creates conditions that lead to higher fire probability (Armenteras et al., 2013; Silva Junior et al., 2018) and biomass reductions have been observed near exposed forest edges (Silva Junior, Aragão, et al., 2020). Regions of higher secondary forests gains in Peru and Colombia are likely associated with local abandonment of agroforestry which covers large areas in the Andes and can lead to regrowth (Aragón et al., 2021). AGC in regions dominated by old‐growth forests showed an increase in the South‐West and decreases in the South‐East Amazon. The South‐West was impacted by the strong drought events of 2005 and 2010 (Saatchi et al., 2013) and showed tree mortality increasing at a faster pace than growth in forest plots within the 1983–2011 period (Brienen et al., 2015). The observed increasing AGC trends over the last decade may therefore indicate a recovery from these past events. Decreasing AGC in the forests of the South‐East is consistent with atmospheric vertical profiling measurements (Gatti et al., 2021). This has been associated with temperature increases and precipitation decreases causing increased tree mortality in an area where background mortality is already high (Brienen et al., 2015; Doughty et al., 2015; Esquivel‐Muelbert et al., 2020).

Spatial patterns are also evident within disagreements between modelled and L‐VOD AGC changes (Figures S4a and S5). Disagreements in areas with high agricultural fraction covers are likely due to non‐forest biomass density changes (Qin et al., 2021) not represented in the model. Greater modelled AGC loss in regions with small‐scale disturbances can be explained by the coarse spatial resolution of the L‐VOD dataset leading to reduced sensitivity, with small changes potentially obscured due to remaining plant water content fluctuations and seasonal flooding (Bousquet et al., 2021; Konings et al., 2021). Nevertheless, the correlation between L‐VOD AGC trends with modelled values (*R* ^2^ = .46) was stronger than previously reported between L‐VOD AGC changes and deforestation or forest cover changes for the Brazilian Amazon (Qin et al., 2021). This is partially because our results include local old‐growth forest variations, though inclusion of degradation processes and secondary forests in addition to deforestation also lead to improved correspondence (Table S6).

### 4.2 Temporal variability of AGC change drivers

The Amazon AGB decreased since 2012 after an increase in 2011 (Figure 3b). The year 2012 marked the lowest observed deforestation activity in the Brazilian Amazon (Instituto Nacional de Pesquisas Espaciais [INPE], 2021), the result of stronger preventative policies put into action in 2004 (Silva Junior, Aragão, et al., 2020). The observed increase, previously reported from L‐VOD data for the Brazilian Amazon, has been attributed to the La Niña event of 2011 following the strong drought year of 2010, and is consistent with independent evidence from forest plots and atmospheric inversions (Qin et al., 2021).

Since 2012, we report an increase in deforestation and associated edge degradation (Figure 3a). Increasing deforestation over this period is supported by national monitoring of forest area loss for the Brazilian Amazon (Instituto Nacional de Pesquisas Espaciais [INPE], 2021) and is related to changes to the Brazilian Forest Code and weaker enforcement of prevention measures (Silva Junior, Aragão, et al., 2020). A recent study reported similar deforestation and edge‐related losses within the 2011–2015 period using the same loss function with Hansen forest change data (Hansen et al., 2013; Silva Junior, Aragão, et al., 2020).

The large degradation losses evident for 2016 (Figure 3, Figure S9) are related to the strong El Niño drought of 2015/2016 resulting in increased fire occurrence (Aragão et al., 2018; Jiménez‐Muñoz et al., 2016; Silva Junior et al., 2019). Large scale forest fires occur mainly due to the fire escaping from recent deforestation or pasture/agriculture management areas (Cano‐Crespo et al., 2015) during extreme drought years in Amazonia (Aragão et al., 2018; Silva Junior et al., 2019).

Old‐growth forest areas showed an increase in AGC in 2015 relative to 2014 (Figure 3a), despite including the onset of the 2015/2016 drought which reduced gross primary productivity in drought‐affected regions. The South Eastern Amazon did show a decrease in AGC which appeared to be compensated by increase in non‐drought affected regions of the South West and increase in the central Amazon (Figure S12) where enhanced growth may be due to greater radiation availability coordinated with flushing of more efficient young leaves at the beginning of the drought (Wu et al., 2016; Yang et al., 2022).

Caution is advised when inferring inter‐annual variations of AGC based on L‐VOD due to the influence of fluctuations in relative water content of vegetation (Konings et al., 2021). In this study we sought to minimize these effects by careful filtering of L‐VOD datasets and comparing data from the wettest periods. Previous efforts to remove water‐content variations from L‐VOD yielded similar AGC change compared with using uncorrected L‐VOD data (Yang et al., 2022). Some remaining variations not due to biomass may remain, increasing the magnitude of anomalies, whereas longer‐term averages and trends remain more reliable.

The average AGC change between 2012 and 2019 suggested a weak source in Amazonian old‐growth forests for this period. A long‐term decline of the Amazon carbon sink until 2011 was previously reported based on forest plots (Brienen et al., 2015) and the extension of this data record will provide more information on the forests current status.

Despite slight increases in secondary forest carbon gains similar to previous reports (Smith et al., 2021) that possibly indicate increasing areas of agricultural abandonment (Aragón et al., 2021), repeated clearing (Heinrich et al., 2021; Nunes et al., 2020), and degradation prevent secondary forests from reaching their full carbon storage capacity (Heinrich et al., 2021) and result in high turnover rates and short residence times (Schwartz et al., 2020; Smith et al., 2021). Recent upturn in deforestation of secondary forest areas casts doubt on their ability to function as longer‐term carbon sinks (Figures S8h and S9) (Smith et al., 2021; Wang et al., 2020).

### 4.3 Country scale differences in processes

Processes driving carbon gains and losses vary between countries due to economic factors and policies (Brito et al., 2019; Walker et al., 2020) but also differences in regional climates, their trends and forest structure (Chen et al., 2015; Fonseca et al., 2017). In Peru, artisanal scale gold mining is a major driver (Espejo et al., 2018) and in Colombia an upturn in conversion of Andean forest to agriculture has previously been observed following the peace agreement between the Colombian Government and FARC (Fuerzas Armadas Revolucionarias de Colombia) in 2012 (Clerici et al., 2020; Murillo‐Sandoval et al., 2021).

Greater losses from degradation than deforestation were observed for smaller Amazon countries (Venezuela, Suriname, Guyana and French Guiana). This is likely associated with a greater proportion of selective logging, typical for countries with lower deforestation rates (Pearson et al., 2014). Comparisons with other datasets (Bullock et al., 2020; Matricardi et al., 2020) show that the degraded area estimates used here (Vancutsem et al., 2021) are likely conservative (Figure S13). A scale‐dependent but consistent definition of forest degradation would greatly aid comparisons of these aggregate drivers of carbon loss as well as facilitate inclusion in carbon budgets, and national inventories.

Brazil's comparatively large AGC losses since 2011 have been evidenced by multiple prior studies (Silva Junior, Aragão, et al., 2020; Smith et al., 2021; Xu et al., 2021), though one shows an AGC increase in the most recent years (Xu et al., 2021). Despite the identified need for Brazil to curb deforestation, Brazil's recent government instead created stimulus for illegal activities such as land‐grabbing, mining and agriculture in indigenous territories and reductions of protected areas (Brito et al., 2019; Rochedo et al., 2018), setting Brazil on a course of forest fragmentation and degradation projected to cause emissions almost 20‐fold higher by 2050 than sustainable scenarios (Assis et al., 2022).

Remaining disagreements in the magnitudes of AGC changes between L‐VOD data and our bottom‐up model highlight that tracking and attribution of AGC changes in the Amazon at national level should be further improved, e.g. by incorporating data from next‐generation biomass mapping sensors (Global Ecosystem Dynamics Investigation (GEDI) (Dubayah et al., 2020), BIOMASS (Le Toan et al., 2011)).

Overall, this study delivers a pessimistic assessment of the current state of the Amazon forest to sequester carbon and mitigate climate change. We deliver further evidence that (i) recent upturns in carbon emissions from deforestation are accompanied by considerable carbon losses through the cascading effects of deforestation including (ii) forest degradation and (iii) high turnover of carbon in regrowing secondary forests while (iv) there is some indication of old‐growth forests becoming a carbon source. Urgent action is therefore required to protect and promote the carbon stocks in the Amazon, the largest contiguous tropical forest on our planet.

## AUTHOR CONTRIBUTIONS

Dominic Fawcett, Stephen Sitch, Luiz E. O. C. Aragão and Philippe Ciais conceptualized and designed the study; Jean Pierre Wigneron, Celso H. L. Silva‐Junior, Viola Heinrich, Christelle Vancutsem, Frédéric Achard, Hui Yang and Xiaojun Li provided datasets, code and/or contributed to the methodology; Dominic Fawcett performed the analysis; Dominic Fawcett wrote the manuscript with inputs from all co‐authors.

## CONFLICT OF INTEREST

The authors declare that they have no competing interests.

## Supporting information


Appendix S1

## Data Availability

The code used to generate the main results of this study is available in the following repository: https://github.com/domfawcett/AmazonLVODCarbon_public_fin (https://zenodo.org/record/7274863#.Y2LB4HbP2Uk). The data to support the results and generate the figures of this manuscript are available at: https://zenodo.org/record/7245450#.Y2QF53bP2Uk. These data were derived from the following resources available in the public domain: Mapbiomas Amazonia C2 (MapBiomas, 2021): https://amazonia.mapbiomas.org; Secondary forest maps (Silva Junior, Heinrich, et al., 2020): https://github.com/celsohlsj/gee_brazil_sv; TMF dataset (Vancutsem et al., 2021): https://forobs.jrc.ec.europa.eu/TMF/download/; ESA CCI Biomass (Santoro & Cartus, 2021): https://doi.org/10.5285/84403d09cef3485883158f4df2989b0c. SMOS‐IC V2 L‐VOD daily data was provided by Jean‐Pierre Wigneron.
