# Supplementary material for: Declining Amazon biomass due to deforestation and subsequent degradation losses exceeding gains
Source: Glob Chang Biol. 2022 Nov 23;29(4):1106–18. doi: 10.1111/gcb.16513 (PMC10100003; doi:10.1111/gcb.16513)
Supplement: Supplementary file 1 — Appendix S1 [file GCB-29-1106-s001.pdf]

Supplementary information for "Declining Amazon biomass due to deforestation and subsequent degradation losses exceeding gains "

D. Fawcett et al.

Supplementary Text

Supplementary Note 1: L-VOD indices and uncertainties in the L-VOD AGC product

The daily L-VOD can be affected by Radio Frequency Interference (RFI), so we first designed strict filtering rules for selecting the "best" quality data from the ascending (ASC) and descending (DESC) orbits according to the root square of the measured and simulated brightness temperature (RMSE-TB). After that, a fit and reconstruction method to derive a smoothed time series from the filtered L-VOD observations least affected by RFI follows the approach described by Thoning, Tans, & Komhyr (1989) originally applied to flask measurements of CO2. We use the C-language implementation provided at https://gml.noaa.gov/ccgg/mbl/crvfit/crvfit.html (accessed 2021.11.24) and apply it to the entire time-series of filtered L-VOD observations per grid-cell. Residuals are filtered using a short-term cut-off of 80 days and long-term cut-off of 667 days. This method yields a smoothed time-series less sensitive to outliers and the long-term trend which excludes seasonality within the data (Fig. S14). As there is little consensus on the "best" L-VOD derived index to represent vegetation we derive three indices sensitive to vegetation biomass in the January-April period of each year: 1. The mean of the smoothed curve, 2. The maximum of the smoothed curve and 3. The mean of the trend curve. The rationale for selecting these is that (1) should be less sensitive to short, potentially spurious maxima, (2) should represent the biomass at maximum plant water content and (3) is less sensitive to seasonal fluctuations in plant water content. By reporting the mean and standard deviation of these three indices we anticipate that the resulting value is more robust and importantly provides a further measure of uncertainty of L-VOD derived AGC changes.

Local anomalies in the L-VOD were identified which led to large, non-feasible changes in biomass. There was a large increase in AGC throughout the Autana region in Colombia in 2016 and large fluctuations of biomass in the vicinity of flooded areas. Grid-cells exhibiting such large changes were filtered out (Fig. S15) using a threshold representing the ~99.9 percentile of annual change values as described in the Methods.

Small differences exist between ASC and DESC data and despite applying thresholds and selecting the more reliable values in terms of TB-RMSE before curve fitting, gaps in the respective datasets resulting from RFI may result in bias for those time periods.

Current efforts on automated L-VOD filtering approaches are expected to reduce the sensitivity of the products to anomalies and overall improve robustness of future L-VOD products.

L-VOD AGC is difficult to independently validate as 1. The coarse spatial resolution of the product adds considerable uncertainty to validation using plot-scale AGC data and 2. Other RS based biomass maps are not independent in that they use similar data to infer biomass in high-biomass areas (Fan et al., 2019). Fan et al. (2019) instead used a bootstrapping and cross-validation method to determine the errors associated with the sampling and calibration of the L-VOD data to AGC and found that these 'internal' errors were an order of magnitude smaller than 'external' errors relating to uncertainties in the reference biomass map, and therefore

negligible. The impact of the combined errors on AGC stocks and changes was estimated to be 20-30% (Fan et al., 2019).

We used the accompanying ESA CCI v2 2017 map of standard errors of biomass estimates and determined that the mean error in AGC per grid cell from L-VOD calibrated using the AGC reference values ±1 SD was 30.6%.

Supplementary Note 2: Differences between L-VOD AGC and modelled AGC

Investigating differences between trends in L-VOD AGC and the modelled values reveals that relative errors are highest in areas with greater agricultural land-cover fractions (2011 extents, Fig. S4). Higher relative errors are expected due to comparatively small AGC within these areas, but the consistently higher decreasing trends in AGC inferred from L-VOD indicate processes not represented by the modelled quantities. For example, L-VOD is also sensitive to non-forest biomass dynamics (Qin et al., 2021). We also infer AGC changes within old-growth forest fractions of each grid cell from L-VOD residual changes in proximal >90% old-growth grid-cells assuming these represent local climatic variations while smaller, more fragmented old-growth patches may show different trajectories (Matricardi et al., 2020). However, the losses due to increased edge area and any visible disturbances were accounted for and in areas with high agricultural fraction these fragmented forest areas account for a smaller proportion of the total AGC.

Total L-VOD AGC for the Amazon (Fig. 3) and individual Amazon countries (Fig. 4) show smaller decreases from 2011 to 2019 than AGC modelled using finer scale land-use and land-cover change (LULCC) datasets.

Part of this difference can be clearly attributed to the large L-VOD inferred increase in AGC in 2011 which is only partially reproduced in the derived old-growth forest response. This can be explained by locally stronger AGC increases within grid-cells that are below the >90% old-growth forest threshold used in this study and may relate to factors including higher productivity in agricultural areas, recovering degraded forest, locally greater secondary forest growth or seasonal flooding in areas not masked by the flood extents used in this study (Bousquet et al., 2021).

While the year 2011 is a La Niña year with conditions resulting in a strong AGC increase, we consider it appropriate to use the L-VOD AGC representing Jan-Apr of this year as a baseline due to the subsequent increase in AGC appearing to mainly affect the values of 2012 (Jan-Apr). We here also report changes per country if 2012 is used as a baseline instead (Fig. S11, Tab. S4) with the main difference being that old-growth forest changes are mostly smaller or negative, resulting in L-VOD AGC being closer to modelled values for Brazil and the entire Amazon.

We further include results of total AGC change per process from an alternative, more conservative estimate of combined uncertainties by using the sum of annual uncertainties per process instead of the root sum of squares (Fig. S16, S17, Tab. S8, S9). These results show greater overlap of error bars with L-VOD based AGC change estimates.

Supplementary Note 3: Uncertainties in LULCC data

The LULCC datasets upon which the reported modelled values of AGC changes through deforestation, degradation and secondary forest growth are based also contain uncertainties from misclassifications, most originating from limited data availability due to frequent cloud cover. Part of the divergence between modelled and L-VOD inferred losses in recent years (Fig. 3b) could be due to uncertainties in identified small-scale disturbances towards the end of the time-series as e.g. the increase in reported larger deforested areas (Instituto Nacional de Pesquisas Espaciais (INPE), 2021) is smaller and a recent bottom-up approach to modelling AGC change in the Amazon shows an increase since 2015 following a decrease (Xu et al., 2021).

However, reported accuracies for the classification datasets are high with 94.1% overall classification accuracy for Mapbiomas collection 2 (MapBiomas, 2021) and 91.4% for detected disturbances reported for the TMF dataset used here to identify degraded forest (Vancutsem et al., 2021).

Uncertainties in automated detection of forest degradation remain. Comparing the TMF dataset to the Bullock, Woodcock, & Souza (2020) disturbance dataset shows similar areas degraded for the Amazon (Fig. S18). On average Bullock et al. (2020) detect 26.6% less new area degraded per year compared to the TMF dataset. It should be noted that the year 2018 of the Bullock dataset which shows a large underestimation was considered less reliable and not used in the study by Bullock et al. (2020). However, both datasets show significantly smaller disturbed areas for the Brazilian Amazon for the 2011-2014 time period compared to the dataset by Matricardi et al. (2020) (266% of TMF and 345% of Bullock degraded area), (Fig. S13). There are clear differences in the methodology used to derive the datasets as Matricardi et al. (2020) make use of a semi-automated approach including manual delineation besides spectral unmixing and textural analysis identification of logged and burned areas based on single Landsat scenes. Automated approaches have the advantage of attributing degradation to individual years but according to this comparison may still underestimate the extent of forest degradation in the Amazon.

Investigating the temporal variation of AGC for areas of intense degradation (Fig. S9 i) revealed disagreement in the year of greatest losses. While modelled losses were highest in 2016, L-VOD shows greater decrease in 2015. Such temporal differences in inter-annual variation originate from delayed detection of disturbed areas due to the low revisit times of Landsat data and frequent cloud cover. In 2015, MODIS burned area (MCD64) shows the most burned area detected in the dry season starting in September, with sustained high values compared to other years in December and January (Fig. S19). These late fires may only be detected by the TMF dataset in 2016, explaining the much higher value for this year (Fig. S18), while being attributed to 2015 in the L-VOD AGC loss calculation (difference between Jan-Apr windows).

Supplementary Figures and Tables

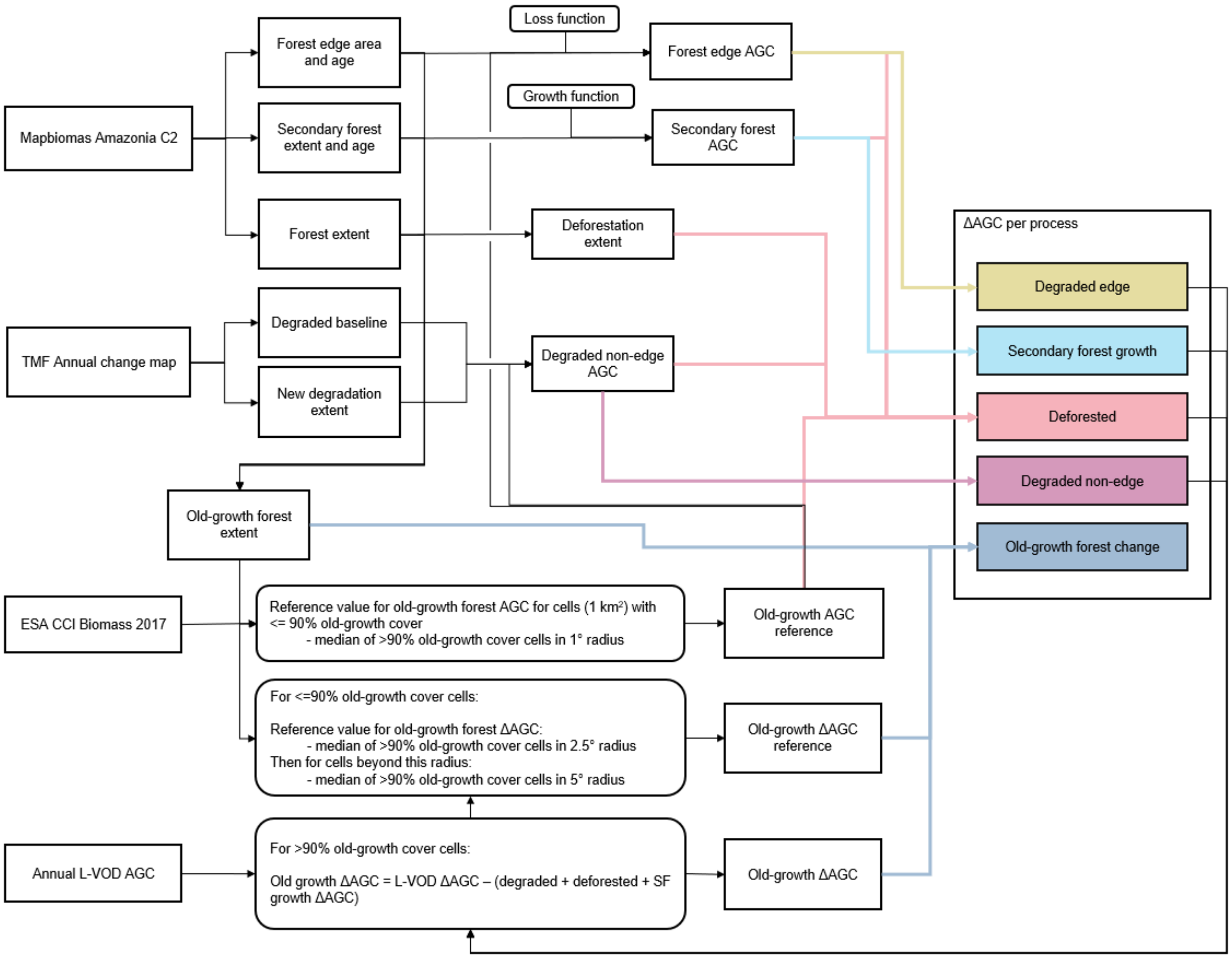

Fig. S1: Flowchart overview of datasets and methodology involved in calculating AGC changes for each process.

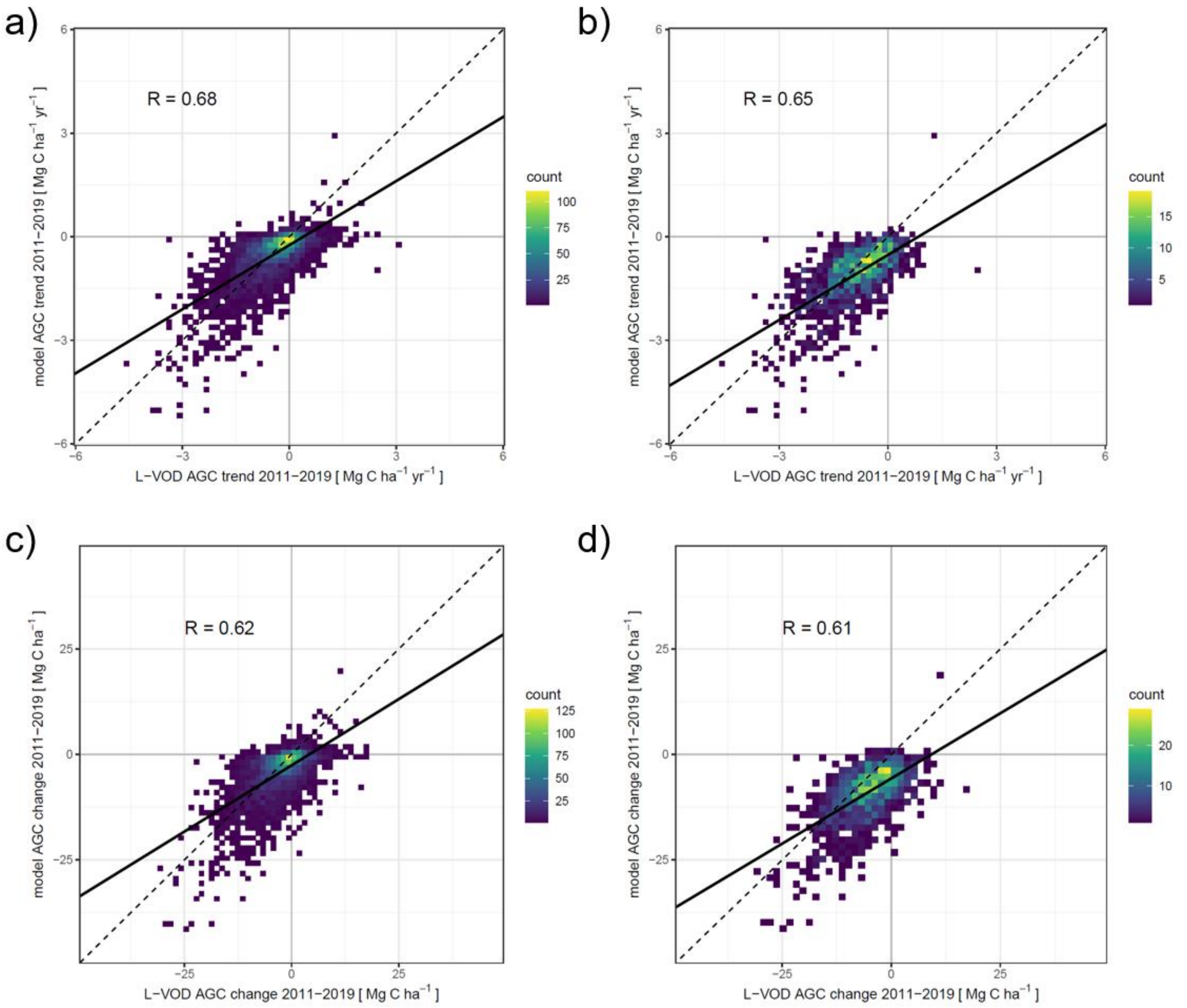

Fig. S2: Scatter plots showing relationship between L-VOD AGC changes and modelled AGC changes. Displayed are trends (a-b) and total changes (c-d) between 2011 and 2019. This relationship was investigated for all grid-cells with <90% old-growth forest (a and c) and grid-cells where old-growth forest equal to >5% of grid-cell area (~40 km^2) was deforested or disturbed within 2011-2019 (b and d).

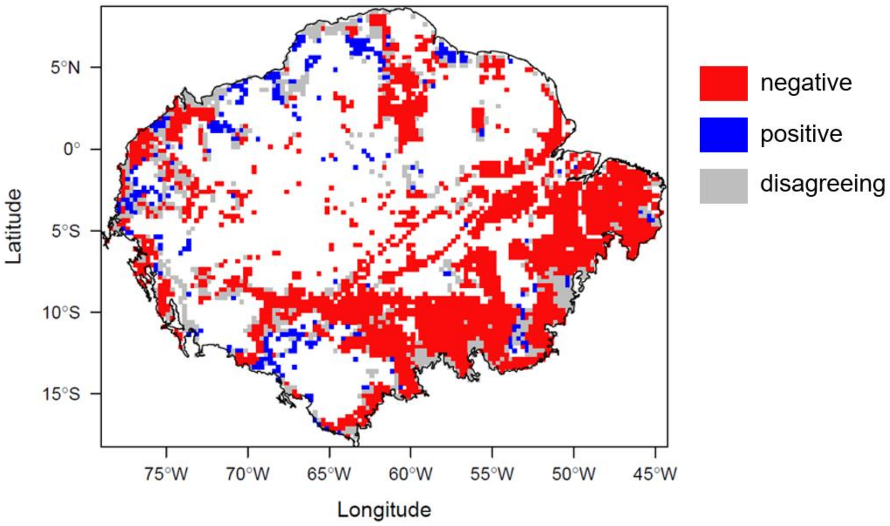

Fig. S3: Agreement and disagreement in AGC trend direction between L-VOD AGC and modelled AGC. Red: Both negative, Blue: Both positive, Grey: Disagreeing.

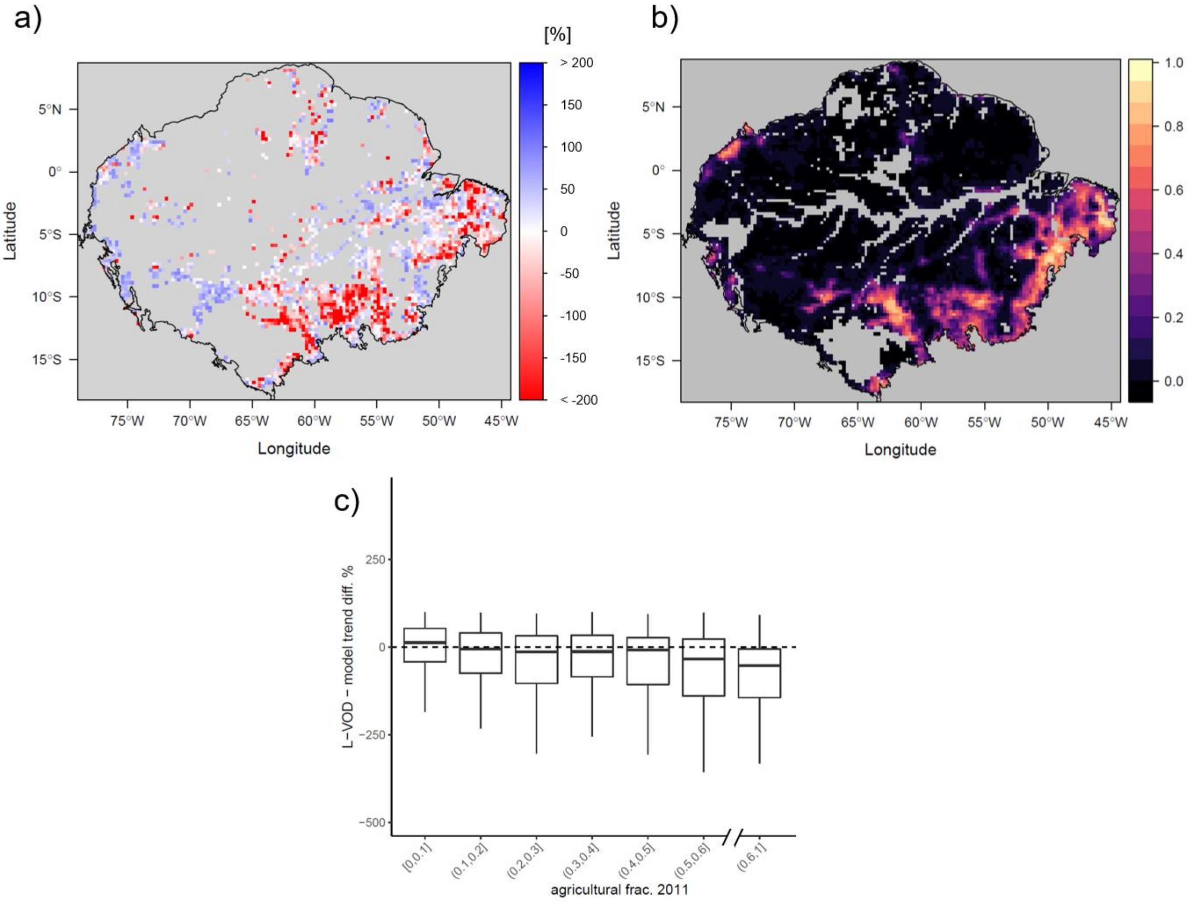

Fig. S4. Differences between L-VOD and modelled trends in relation to agricultural fraction. a) relative difference between L-VOD AGC and modelled trend slopes for AGC-loss grid cells. b) agricultural land-cover fraction in 2011 per grid-cell from Mapbiomas C2. c) relative difference between L-VOD AGC and modelled trend slopes for bins of varying agricultural fractional cover. There was a significant negative correlation at the 95% confidence

level. The bold line indicates the median, boxes indicate the interquartile range and whiskers extend to the extreme value within 1.5 times the interquartile range. Outliers beyond this range are omitted for visualisation purposes.

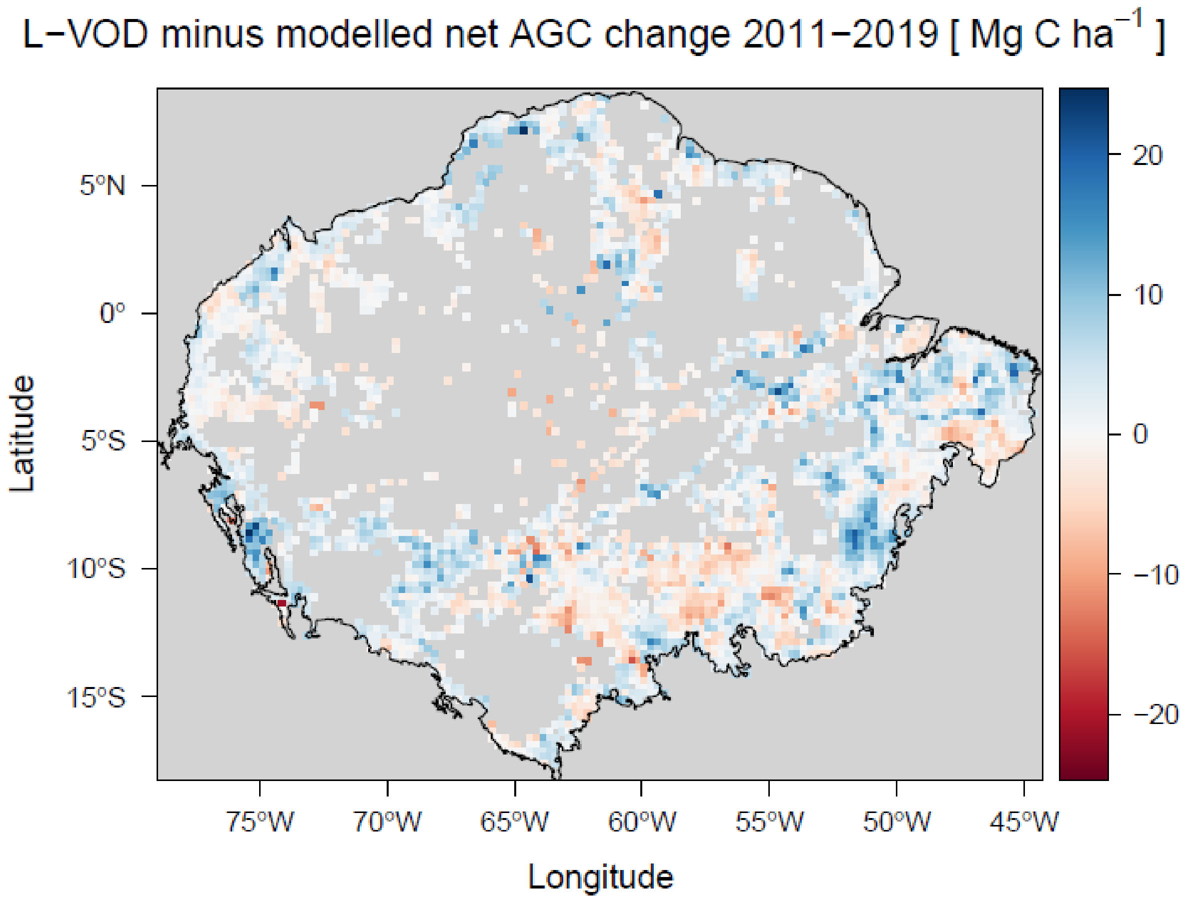

Fig. S5. Total difference between L-VOD AGC change and modelled AGC change for each 0.25° grid-cell from the beginning of 2011 to the beginning of 2019. Cells with >90% old-growth forest cover in 2018 were excluded. Positive (blue) cells indicate smaller losses or greater gains inferred by L-VOD AGC compared to the modelled values, negative (red) areas indicate greater losses or smaller gains inferred by L-VOD AGC compared to the modelled values.

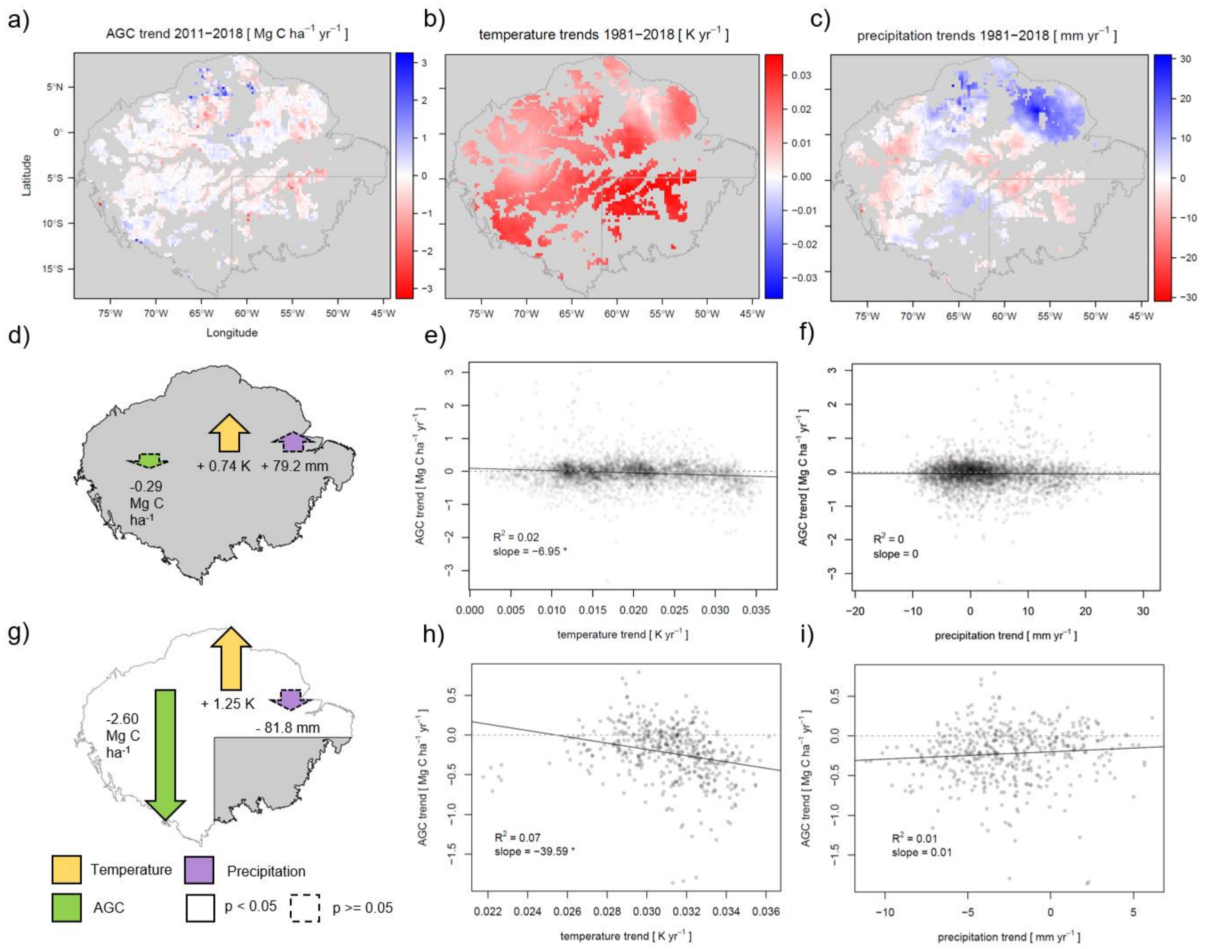

Fig. S6: Trends in AGC, precipitation and temperature for grid-cells covered by >90% old-growth forest and their correlations. a) Trends in L-VOD AGC (Mg C ha^{-1} yr^{-1}, Jan-Apr 2011 to Jan-Apr 2019), b) trends in mean annual temperature (K yr^{-1}, 1981-2018), c) trends in total annual precipitation (mm yr^{-1}, 1981-2018), d) total change inferred from trends in AGC (2011-2019), temperature (1981-2018) and precipitation (1981-2018) for >90% old-growth forest grid-cells for the entire Amazon biome, e-f) correlation of AGC trends for individual grid cells with trends in temperature (e) and precipitation (f). g) total changes for >90% old-growth forest grid-cells in the South-East of the Amazon biome, h-i) correlation of AGC trends for individual grid cells with trends in temperature (h) and precipitation (i). Trends in AGC are derived from L-VOD AGC data, trends in temperature and precipitation are derived from the ERA 5 Land ECMWF Climate Reanalysis dataset.

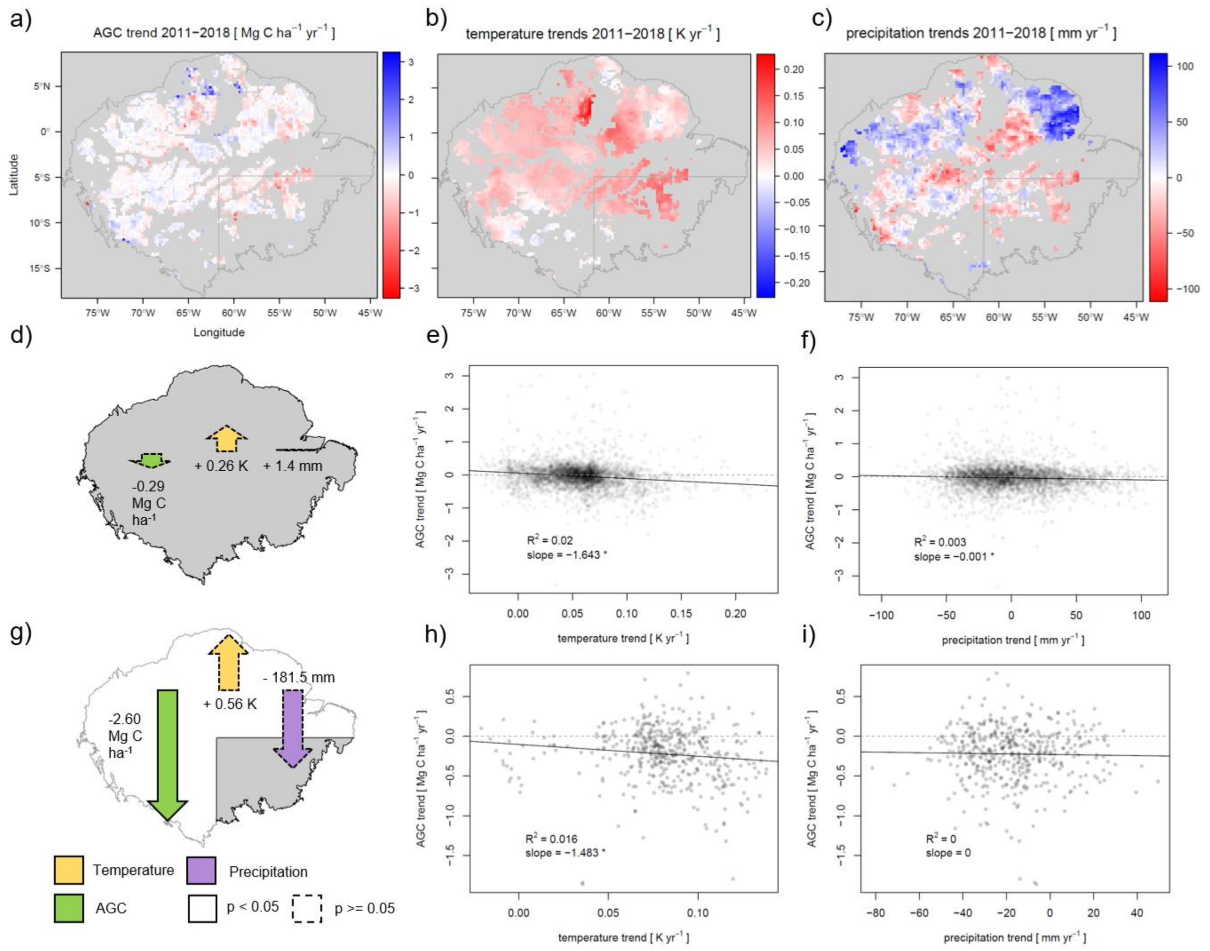

Fig. S7: Trends in AGC, precipitation and temperature for grid-cells covered by >90% old-growth forest and their correlations. a) Trends in L-VOD AGC (Mg C ha^{-1} yr^{-1}, Jan-Apr 2011 to Jan-Apr 2019), b) trends in mean annual temperature (K yr^{-1}, 2011-2018), c) trends in total annual precipitation (mm yr^{-1}, 2011-2018), d) total change inferred from trends in AGC (2011-2019), temperature (2011-2018) and precipitation (2011-2018) for >90% old-growth forest grid-cells for the entire Amazon biome, e-f) correlation of AGC trends for individual grid cells with trends in temperature (e) and precipitation (f). g) total changes for >90% old-growth forest grid-cells in the South-East of the Amazon biome, h-i) correlation of AGC trends for individual grid cells with trends in temperature (h) and precipitation (i). Trends in AGC are derived from L-VOD AGC data, trends in temperature and precipitation are derived from the ERA 5 Land ECMWF Climate Reanalysis dataset.

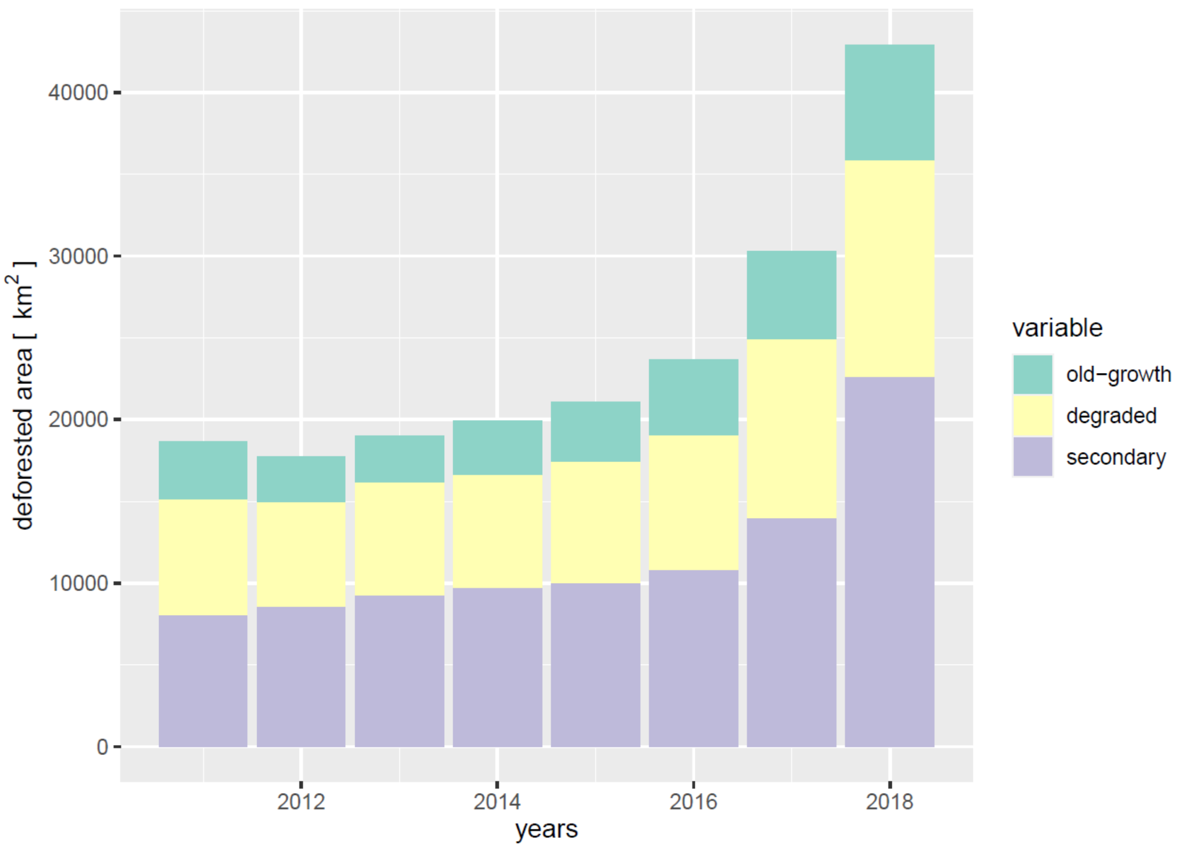

Fig. S8: Area lost through deforestation each year in the entire Amazon biome for old-growth, degraded and secondary forest. Classes are based on annual Mapbiomas C2 change from forest to non-forest areas. Degraded forest includes forest edges.

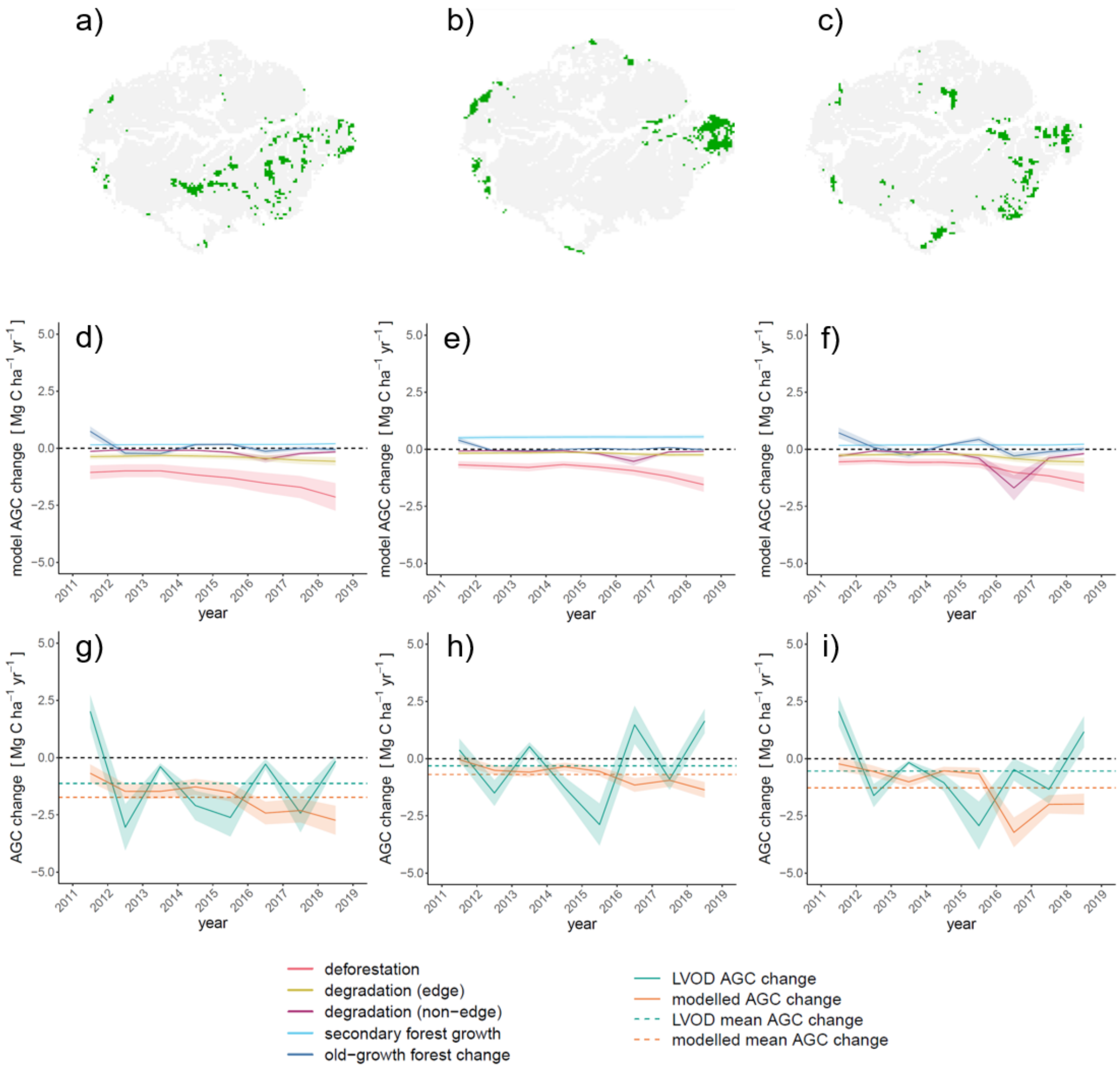

Fig. S9. Analysis of spatial subsets dominated by specific processes. Spatial subsets representing the 95th percentile of cumulative a) deforestation, b) degradation and c) mean secondary forest fractions along with their modelled AGC change per year associated with processes of deforestation, degradation (edge and non-edge), secondary forest growth and old-growth forest change (d-f) and the net AGC change per year compared to L-VOD AGC change (g-i). Ribbons represent uncertainties associated with the ESA CCI biomass map (± 1 SD, for deforestation, edge and non-edge degradation loss), from the secondary forest growth model (± 1 SD of average growth rate) and L-VOD AGC uncertainties reported for old-growth forest change inferred from ± 1 SD of the ESA CCI biomass map used for calibration and the three L-VOD indices (see Methods).

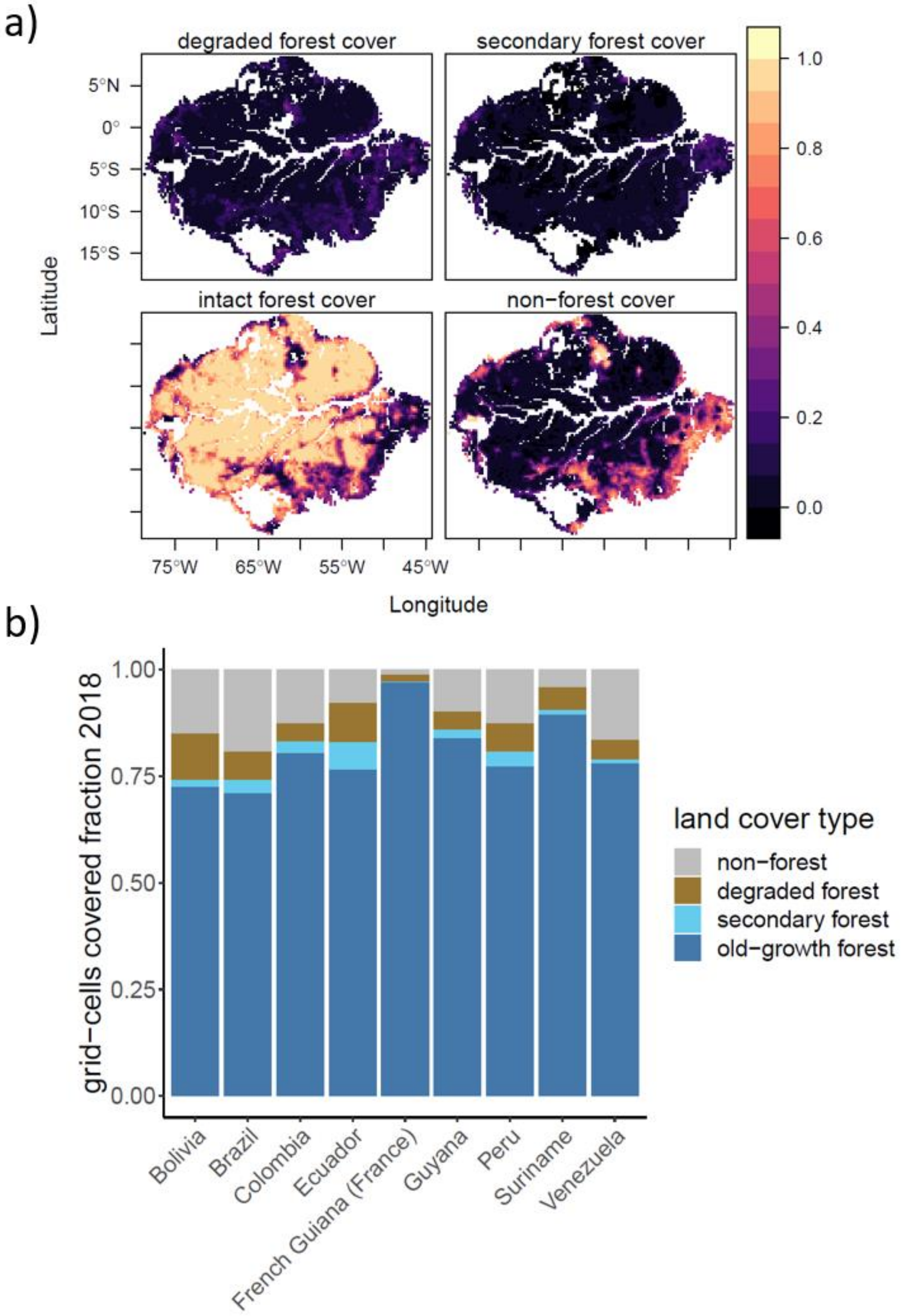

Fig. S10: Visualisation of the extent of forest cover types for the studied area, shown a) spatially as fraction of grid-cell area and b) aggregated by grid-cells belonging to each of the Amazon countries.

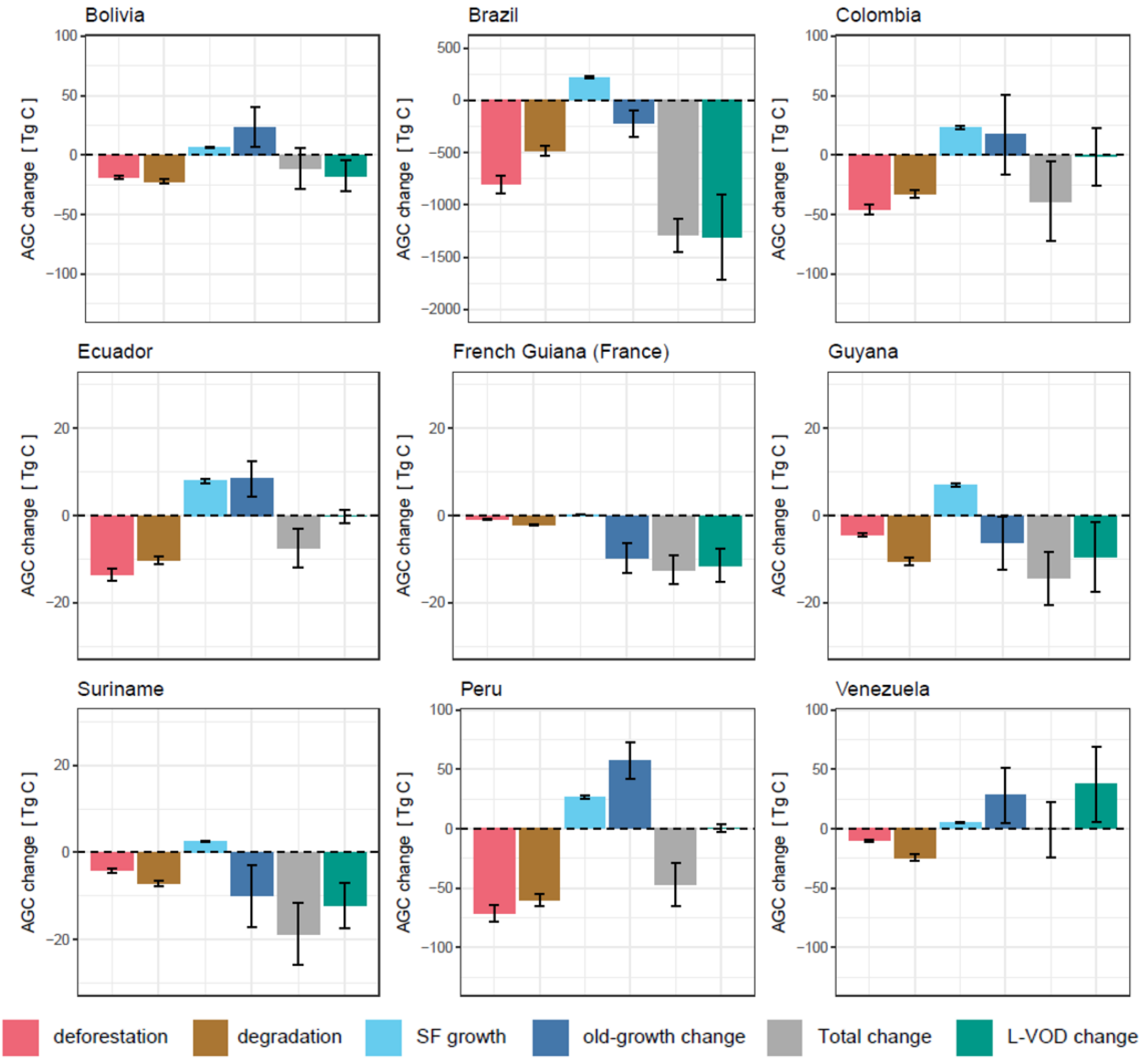

Fig. S11: AGC change (2012 to 2019) associated with different processes (deforestation, degradation, secondary forest growth, old-growth forest change), combined and L-VOD inferred AGC change for parts of the Amazon forest divided by country. Note the different y-axes to visualize changes for smaller countries. Whiskers represent uncertainties associated with the ESA CCI biomass map (± 1 SD, for deforestation, edge and non-edge degradation loss), from the secondary forest growth model (± 1 SD of average growth rate) and uncertainties reported for old-growth forest change and L-VOD change inferred from ± 1 SD of the ESA CCI biomass map used for calibration of L-VOD AGC and the three L-VOD indices (see Methods). The total modelled changes include the combination of these uncertainties.

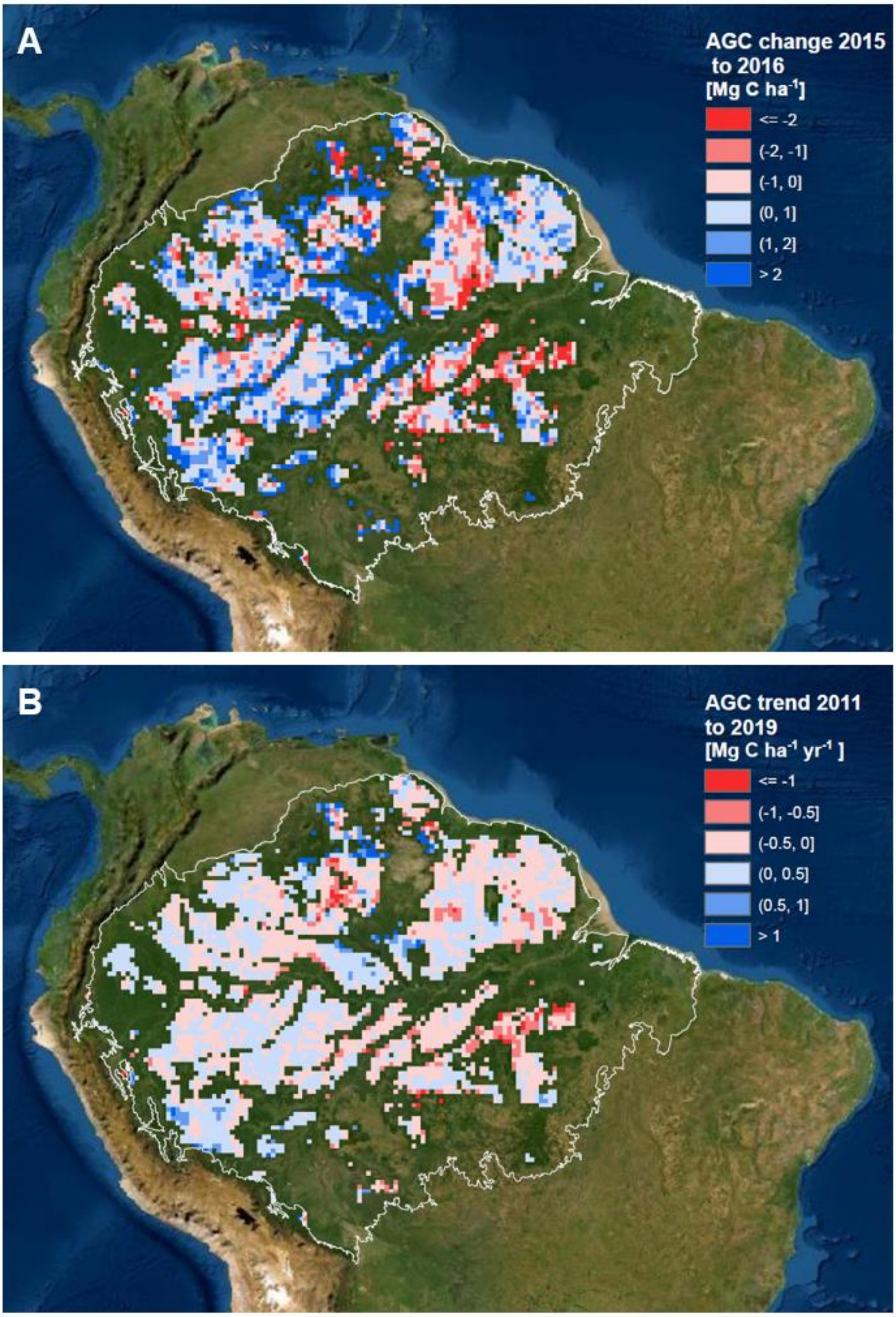

Fig. S12: a) L-VOD AGC changes in old-growth forests from 2015 to 2016. Spatial patterns in AGC changes over the Amazon biome highlighted for >90% old-growth forest covered grid-cells. b) AGC trends over 2011 – 2019 for the same grid-cells.

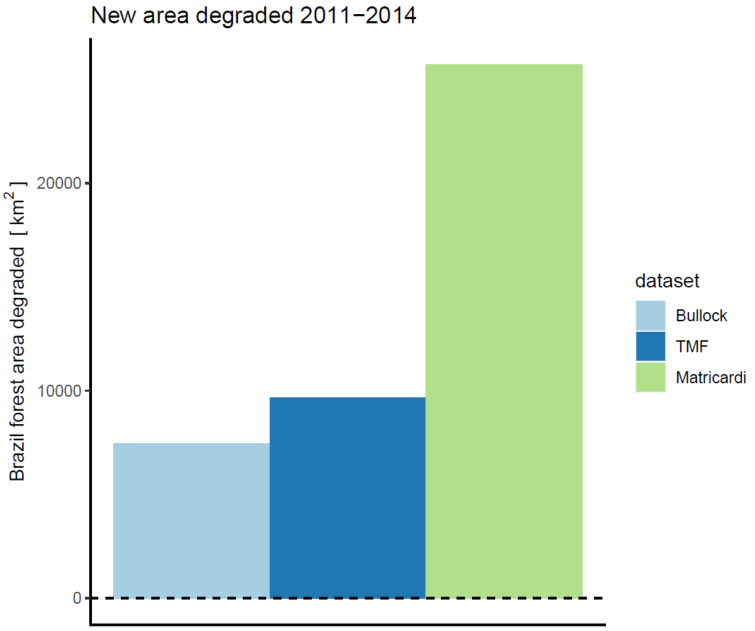

Fig. S13: Total new area of the Brazilian Amazon degraded in the 2011-2014 period inferred from the Bullock et al. (2020) **dataset, TMF** (Vancutsem et al., 2021) **and Matricardi et al.** (2020) **datasets.** From the Matricardi dataset we include burned areas and logged areas, the Bullock and TMF datasets include the summed annually detected degradation. Forest edge degradation was excluded from all datasets.

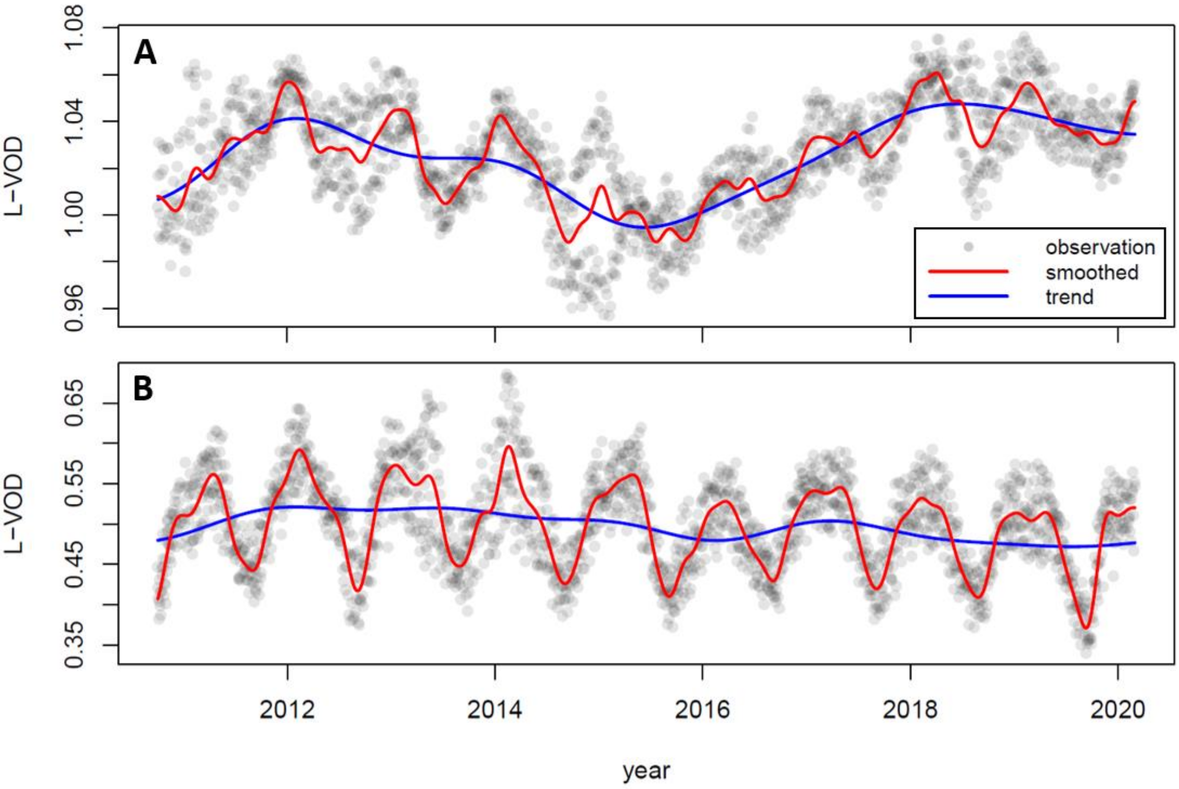

Fig. S14: Filtered L-VOD observations, smoothed data and trend curve illustrated for a) an old-growth forest (high biomass) grid-cell with weak L-VOD seasonality and b) a partially deforested grid-cell (medium biomass) with strong L-VOD seasonality.

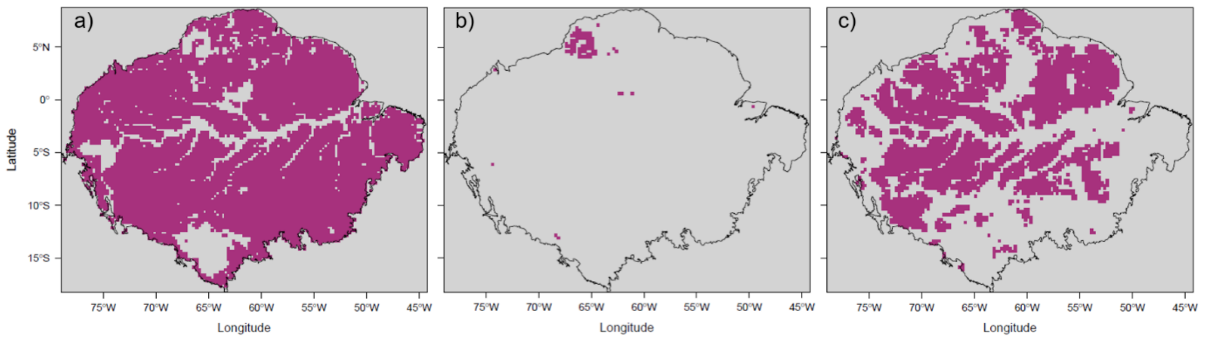

Fig. S15: Visualization of L-VOD data availability. a) L-VOD data availability for the Amazon biome post filtering and masking. Grid-cells affected by water, extreme topography and local biomass anomalies were excluded. b) Grid-cells excluded due to max. annual changes >20 Mg C ha^{-1} (~99.9 percentile of all annual change values) c) Grid-cells with L-VOD data availability with >90% old-growth forest cover in 2018.

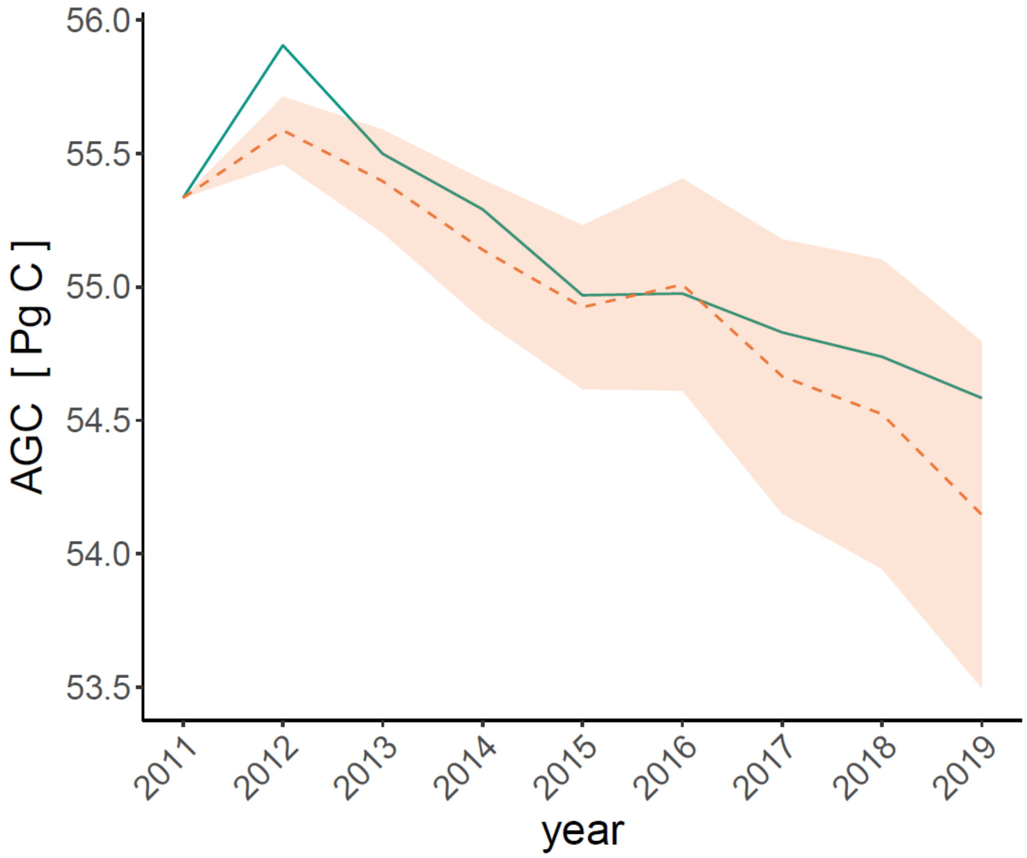

Fig. S16: Total AGC from 2011 to 2019 over the Amazon, modelled and inferred from L-VOD, where values represent AGC at the beginning of the respective year (Jan-Apr). Alternative version to Fig. 3 b) with total uncertainties for each year resulting from summation instead of root sum of squares**.**

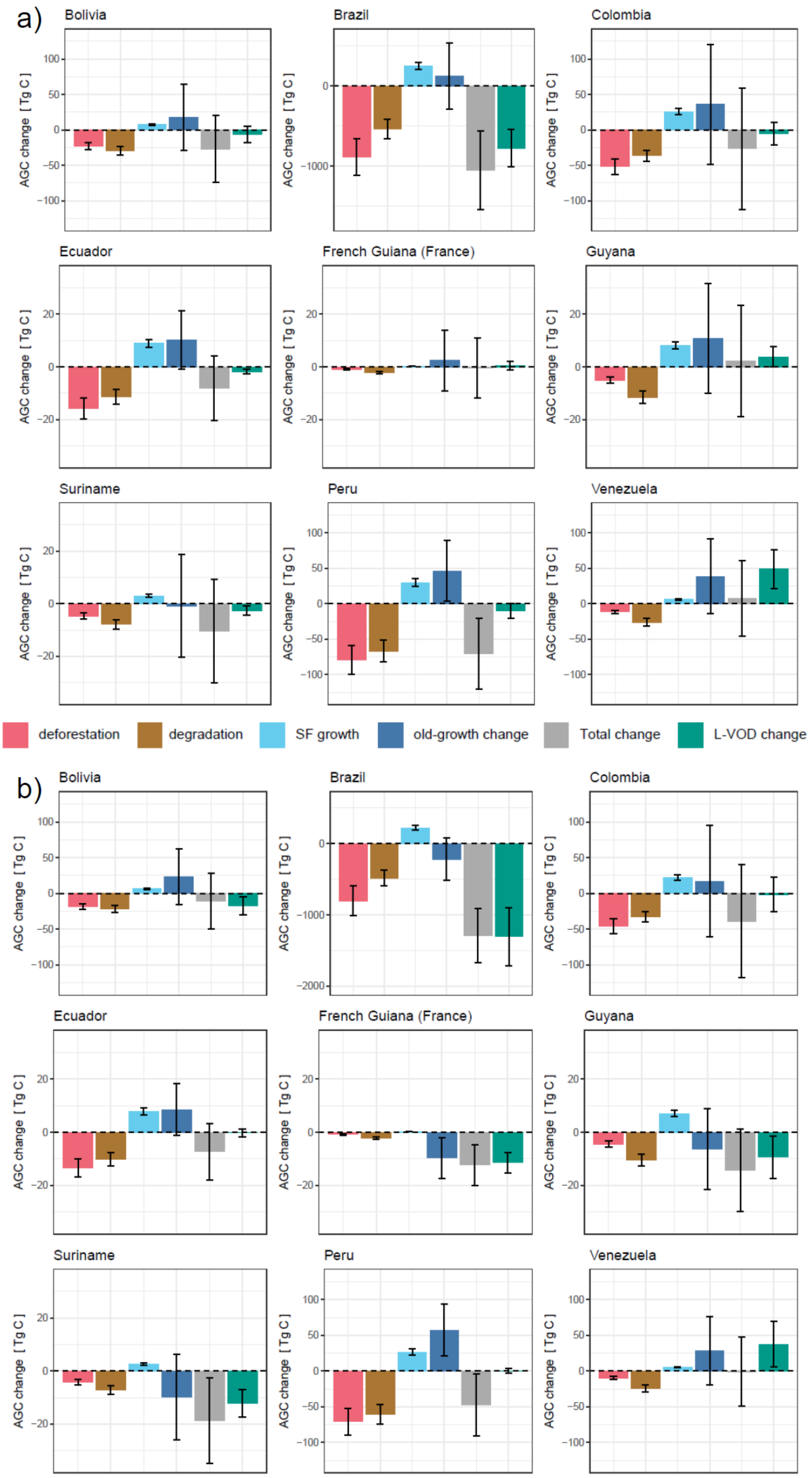

Fig. S17: Country scale AGC changes in the Amazon forest a) from 2011 to 2019, b) from 2012 to 2019. Alternative versions to Fig. 4 and Fig. S6 with annual uncertainties combined per process using summation instead of root sum of squares.

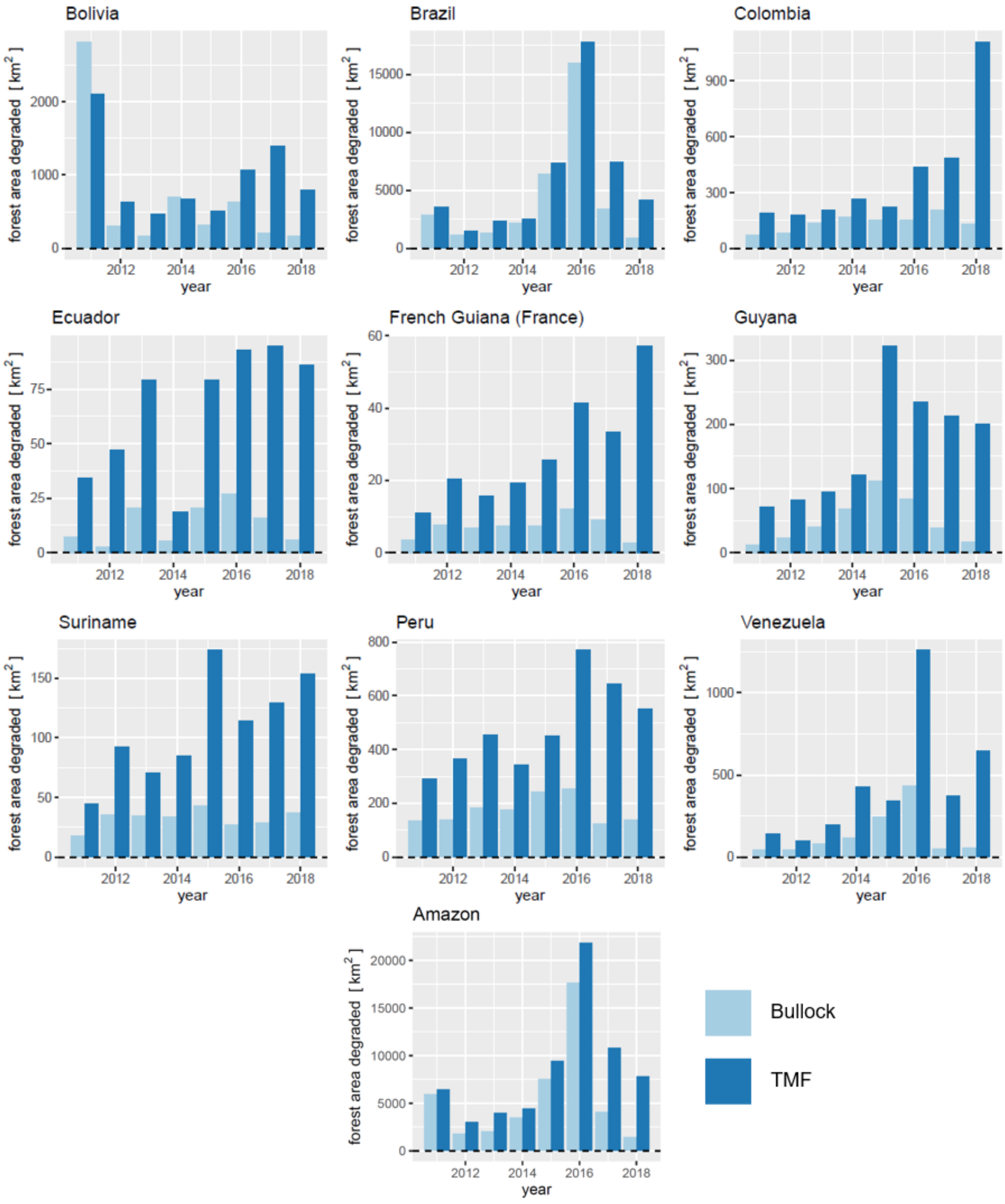

Fig. S18: New Amazon forest area degraded per year and country inferred from the Bullock et al. (2020) **and TMF** (Vancutsem et al., 2021) **datasets. They include the annually detected degradation, excluding forest edges.**

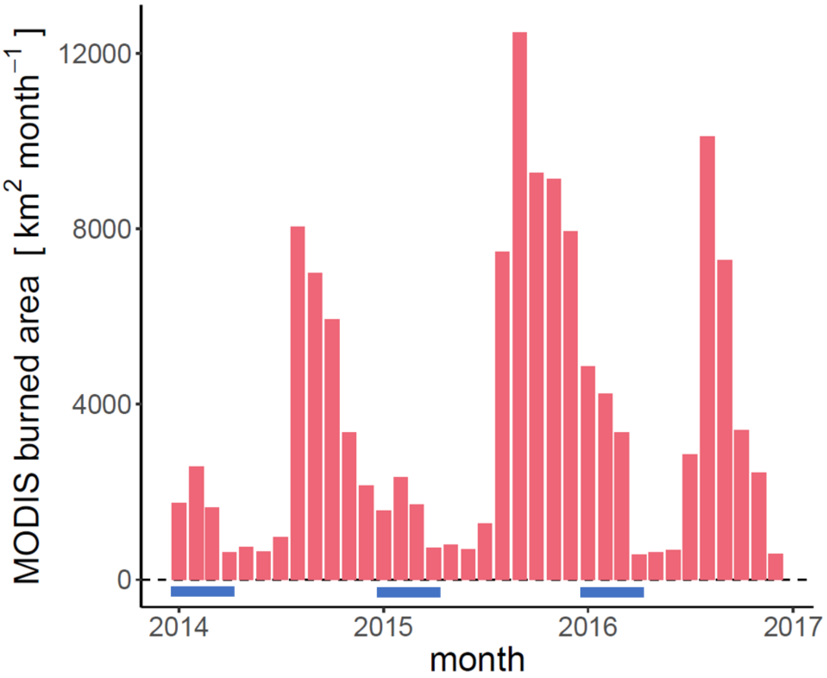

Fig. S19: MCD64A1v6 monthly burned area for all studied grid-cells in 2014 to 2017. Blue bars indicate the four-month time-windows used for L-VOD AGC calculations.

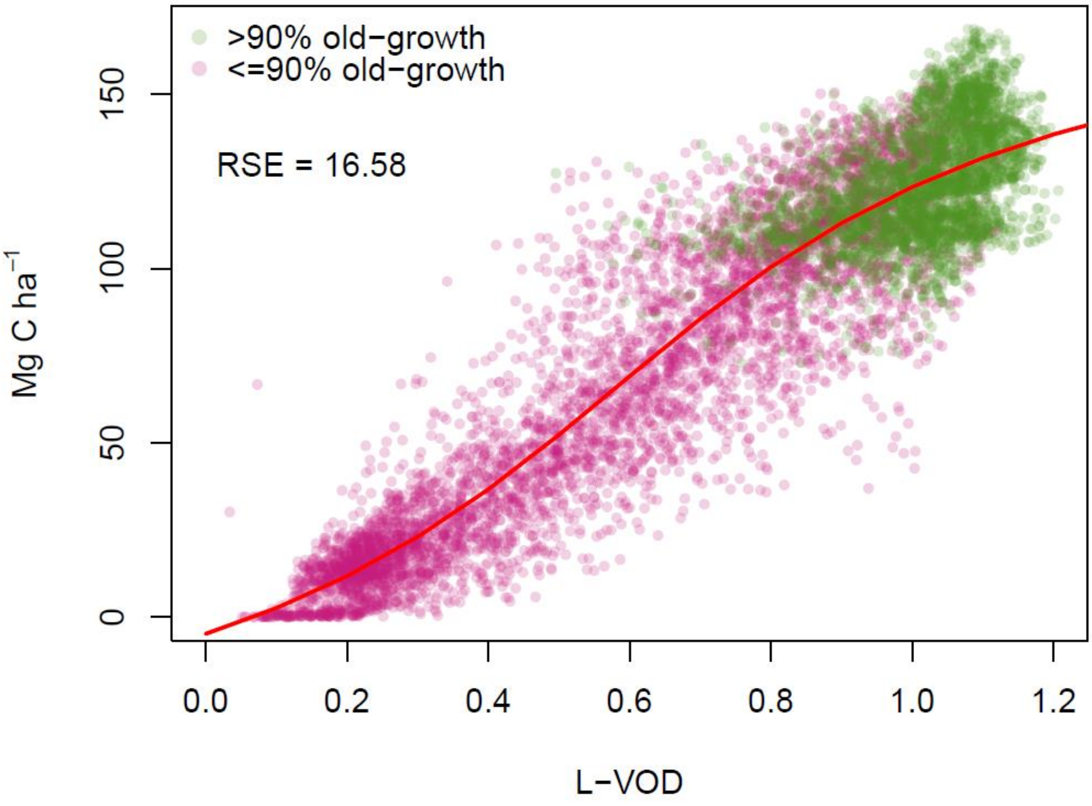

Fig. S20: Fitted four-parameter function (Fan et al., 2019) **relating the 'trend mean' L-VOD index values for 2017 to AGC from the ESA CCI 2017 biomass map, indicating >90% old-growth forest covered cells**

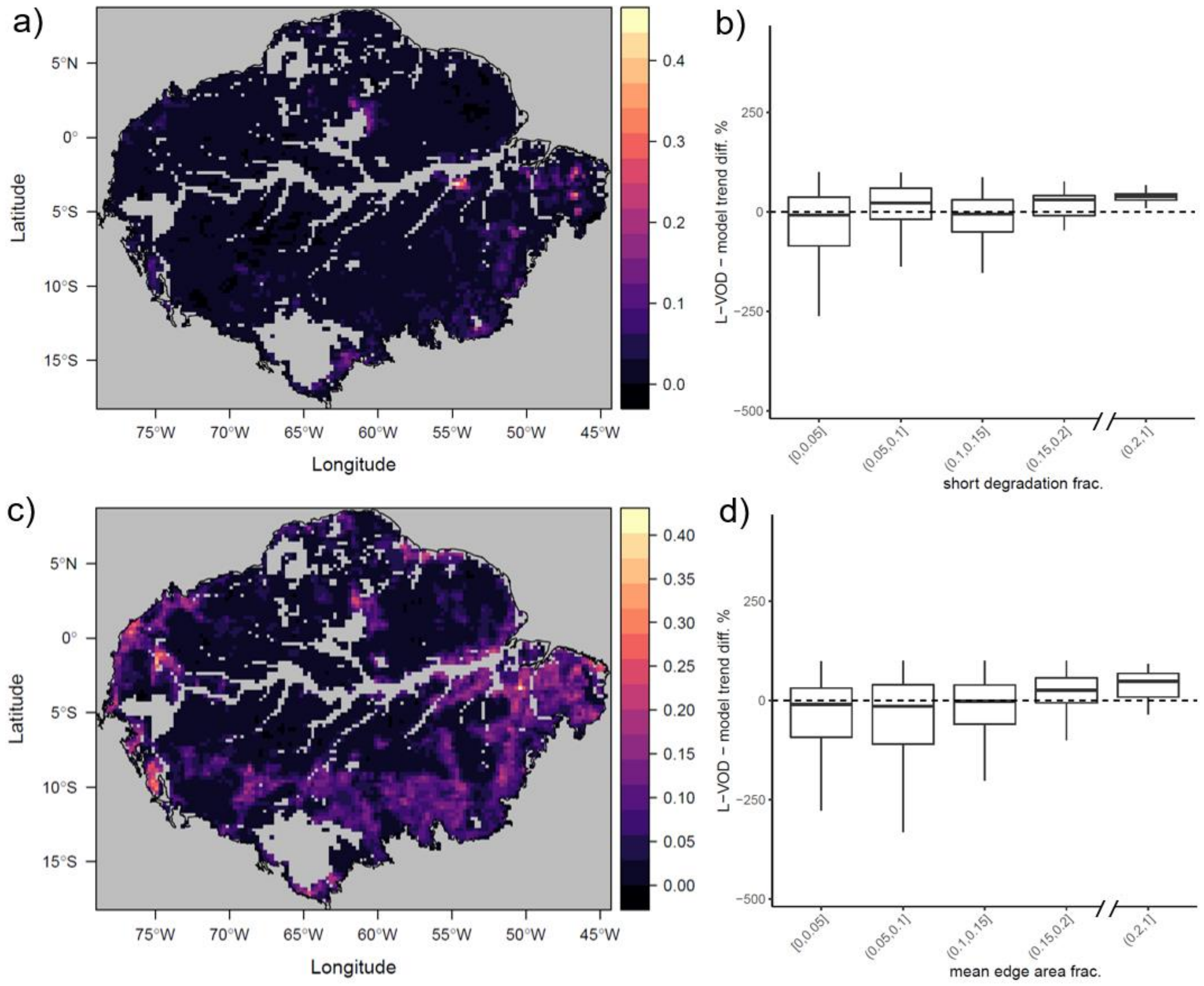

Fig. S21: a) fraction of grid-cell that experienced short-term degradation events (e.g. logging, burning) (Vancutsem et al., 2021)**, b) relative difference between L-VOD AGC and RS modelled trend slopes for bins of varying short-term degradation fractions.** There was a significant positive correlation at the 95% confidence level. c) Fraction of grid-cell covered by anthropogenic forest edge experiencing degradation (120 m from forest edge, mean 2011-2018), d) relative difference between L-VOD AGC and RS modelled trend slopes for bins of varying forest edge fractions. There was a significant positive correlation at the 95% confidence level. The bold line indicates the median, boxes indicate the interquartile range and whiskers extend to the extreme value within 1.5 times the interquartile range. Outliers beyond this range are omitted for visualisation purposes.

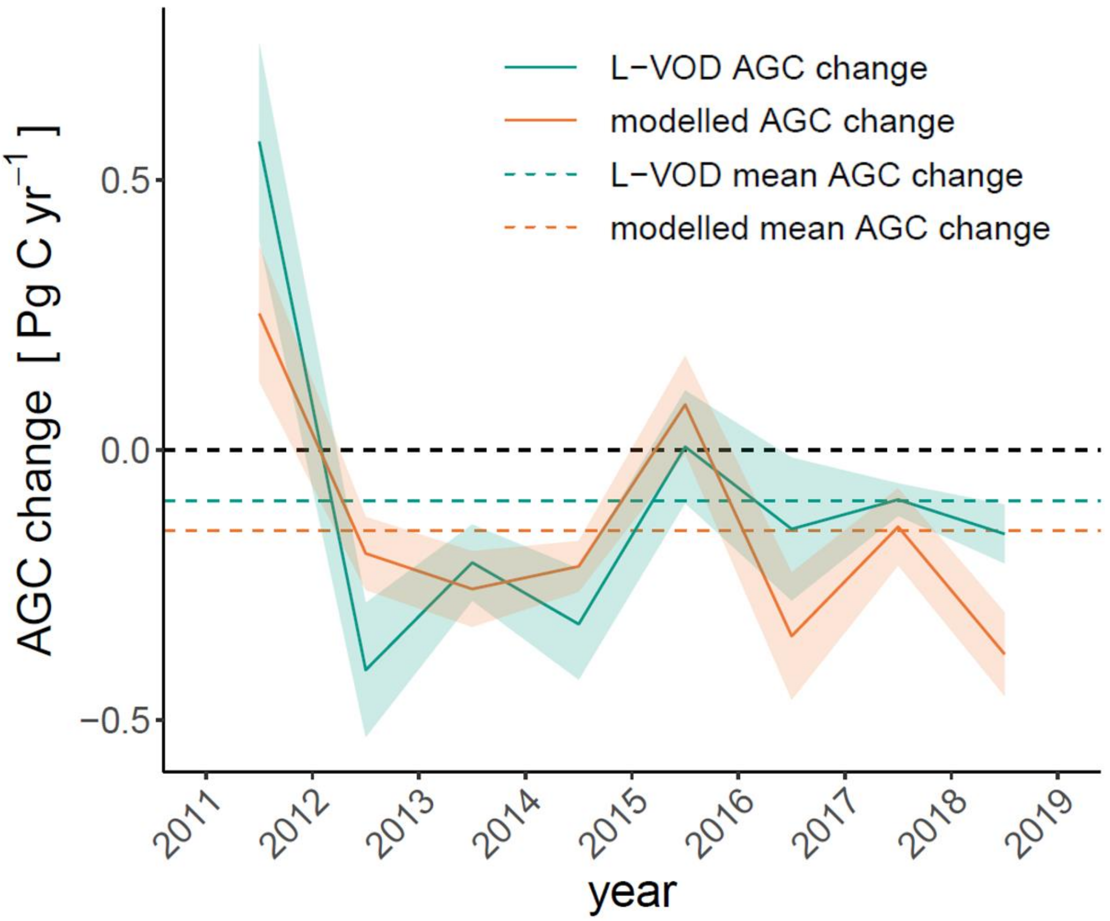

Fig. S22: L-VOD and RS modelled Amazon AGC change and mean change per year. Ribbons represent combined uncertainties associated with the ESA CCI biomass map (± 1 SD, for deforestation, edge and non-edge degradation loss), from the secondary forest growth model (± 1 SD of average growth rate) and L-VOD AGC uncertainties reported for old-growth forest change and L-VOD AGC change inferred from ± 1 SD of the ESA CCI biomass map used for calibration and the three L-VOD indices (see Methods).

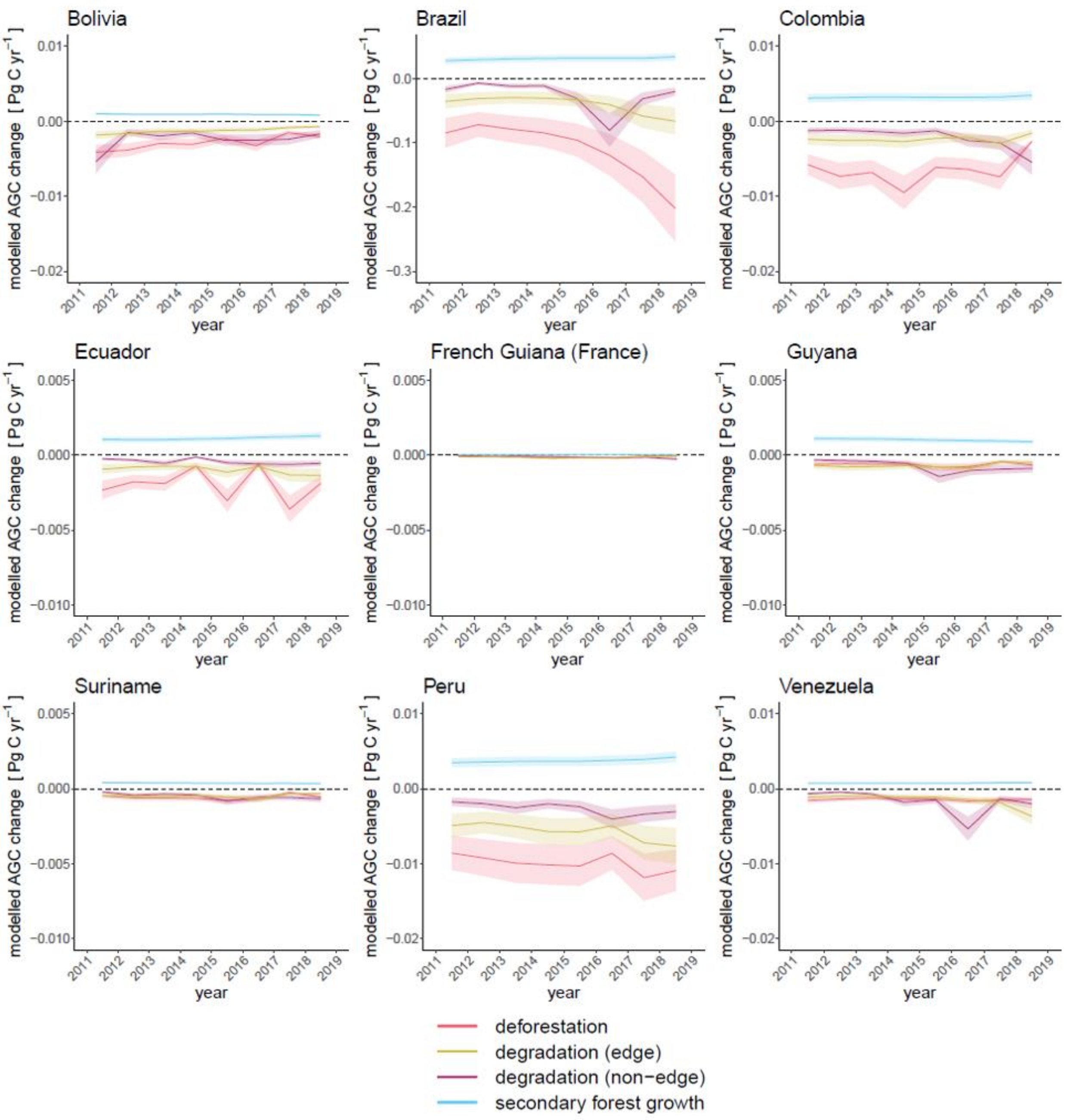

Fig. S23: RS modelled AGC change per process for all Amazon countries (note different y-axis scales to visualize changes for smaller countries), excluding old-growth forest variations. Ribbons represent uncertainties associated with the ESA CCI biomass map (± 1 SD, for deforestation, edge and non-edge degradation loss), from the secondary forest growth model (± 1 SD of average growth rate).

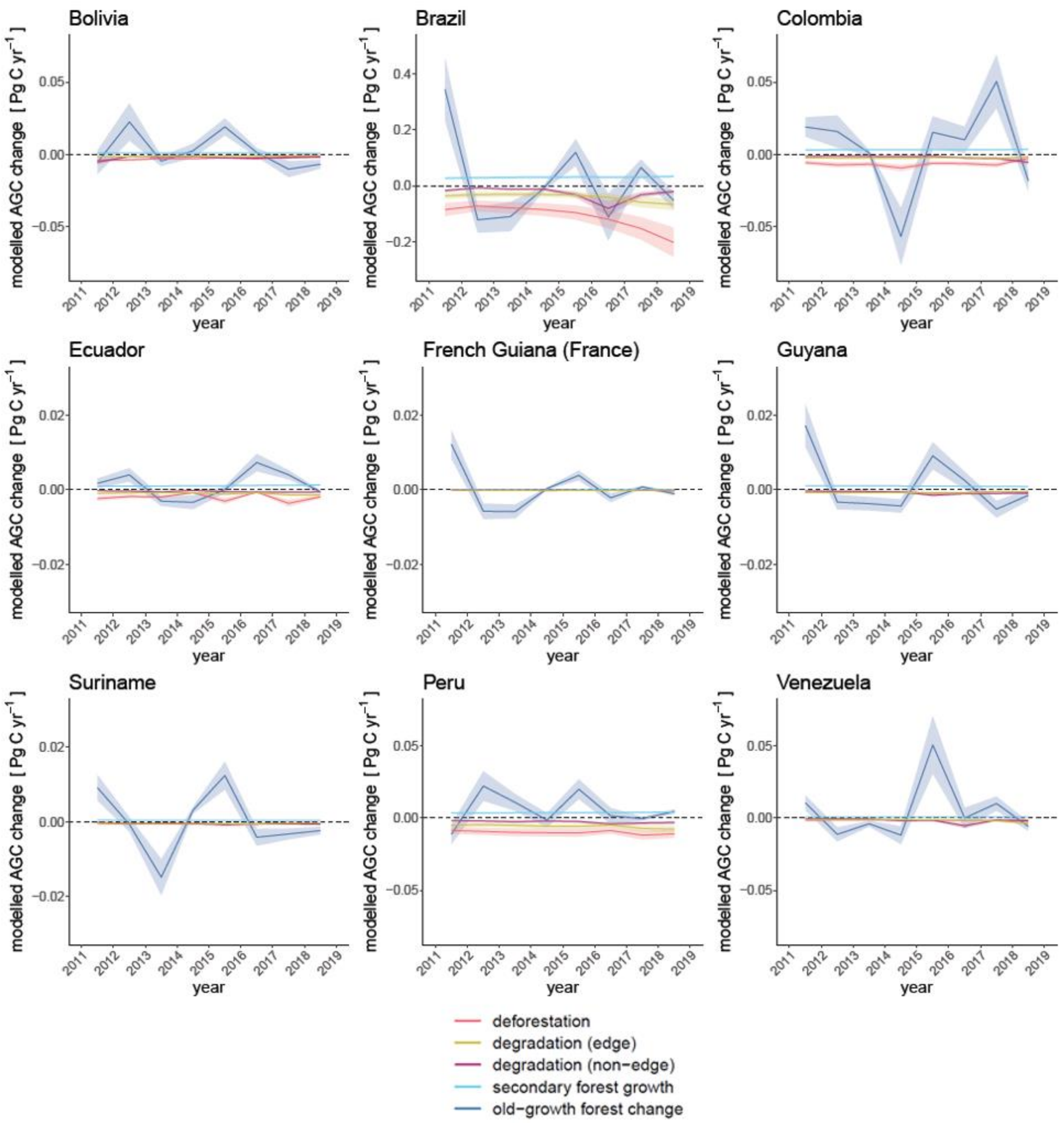

Fig. S24: RS modelled AGC change per process for all Amazon countries (note different y-axis scales to visualize changes for smaller countries) and L-VOD inferred old-growth forest changes. Ribbons represent uncertainties associated with the ESA CCI biomass map (± 1 SD, for deforestation, edge and non-edge degradation loss), from the secondary forest growth model (± 1 SD of average growth rate) and L-VOD AGC uncertainties reported for old-growth forest change inferred from ± 1 SD of the ESA CCI biomass map used for calibration and the three L-VOD indices (see Methods).

Table S1: Values of a-d of the curves fitted to the three different L-VOD indices (Equation 1 of the main manuscript), with their standard errors in brackets.

L-VOD index	*a*	*b*	*c*	*d*
Smoothed mean	184.08 (3.81)	2.44 (0.10)	0.60 (0.01)	-2.98 (1.04)
Smoothed max	183.66 (3.65)	2.56 (0.10)	0.63 (0.01)	-3.36 (1.03)
Trend mean	193.85 (4.53)	2.15 (0.10)	0.58 (0.01)	-4.81 (1.10)

Tab. S2: Functions for calculating (1) the AGC of secondary forest based on age *t*, (2) forest edge AGC loss based on age *t* and (3) the relation between old-growth and degraded forest AGC using a constant factor.

Quantity	Function		Reference
Secondary forest AGC	$AGC_t = 122.5\,(1 - e^{-0.037t})^{1.085}$	(1)	(Heinrich et al., 2021)
Forest edge biomass loss	$AGC_{loss} = \frac{-42.815t}{0.836 + t}$	(2)	(Silva Junior et al., 2020)
Degraded forest biomass (non-edge)	$AGC_{degraded} = 0.6468 AGC_{old-growth}$	(3)	ESA CCI 2017 and TMF map (Santoro & Cartus, 2021; Vancutsem et al., 2021)

Tab. S3: Net changes in AGC for Amazon countries from 2011 to 2019 as modelled for each process using land-cover data, ESA CCI biomass map and L-VOD AGC (old-growth forest change). Uncertainties that arise from the ESA CCI biomass map (± 1 SD, for deforestation, edge and non-edge degradation loss) and from the secondary forest growth model (± 1 SD of average growth rate) are reported. Uncertainties for old-growth forest change represent ± 1 SD of the different L-VOD indices and the ESA CCI biomass map used for calibration. The total modelled change includes the combination of these uncertainties.

				Modelled net changes 2011-2019 [Tg C]												[% Tg C]	
Country	N cells	AGC 2011		Deforestation loss		Edge loss		Degradation loss (non-edge)		Secondary forest growth		Old-growth forest change		Total modelled change		Total modelled relative change	
Bolivia	268	1 869.56	±568.6	-22.93	±1.9	-9.93	±1.0	-19.33	±2.2	7.45	±0.4	17.85	±18.8	-26.89	±19.0	-1.44	±1.0
Brazil	4 717	35 163.42	±10 705.2	-889.48	±87.4	-325.65	±37.7	-210.95	±30.6	247.34	±14.9	123.90	±168.8	-1 054.84	±196.8	-3.00	±0.6
Colombia	586	4 659.27	±1 418.4	-51.94	±4.3	-19.00	±2.1	-17.44	±2.2	25.81	±1.6	36.10	±33.9	-26.47	±34.4	-0.57	±0.7
Ecuador	92	836.86	±254.7	-15.88	±1.6	-7.87	±0.9	-3.57	±0.4	8.95	±0.5	10.23	±4.2	-8.15	±4.6	-0.97	±0.6
French Guiana	95	888.09	±270.6	-0.93	±0.1	-1.18	±0.1	-1.09	±0.1	0.30	±0.0	2.43	±5.1	-0.46	±5.1	-0.05	±0.6
Guyana	229	1 947.29	±593.5	-5.09	±0.5	-5.58	±0.6	-5.97	±0.7	8.06	±0.5	10.89	±8.3	2.30	±8.4	0.12	±0.4
Suriname	157	1 431.40	±436.2	-4.64	±0.4	-3.80	±0.4	-4.05	±0.5	3.00	±0.2	-0.84	±7.9	-10.33	±7.9	-0.72	±0.6
Peru	626	5 697.31	±1 734.8	-79.55	±7.3	-45.75	±5.1	-21.20	±2.4	29.99	±1.8	46.23	±17.0	-70.29	±19.4	-1.23	±0.3
Venezuela	391	2 836.45	±865.7	-11.53	±0.9	-12.22	±1.5	-13.81	±1.9	6.05	±0.4	38.67	±23.9	7.17	±24.0	0.25	±0.8
Amazon	7 162	55 333.55	±16 847.9	-1 081.99	±102.5	-431.01	±48.9	-297.41	±39.4	336.96	±20.3	285.50	±214.7	-1 187.94	±246.9	-2.15	±0.4

Table S4: Net changes in AGC for Amazon countries from 2012 to 2019 as modelled for each process using land-cover data and the total change in AGC from L-VOD. Uncertainties that arise from the ESA CCI biomass map (± 1 SD, for deforestation, edge and non-edge degradation loss) and from the secondary forest growth model (± 1 SD of average growth rate) are reported. Uncertainties for old-growth forest change represent ± 1 SD of the different L-VOD indices and the ESA CCI biomass map used for calibration. The total modelled change includes the combination of these uncertainties. Uncertainties for the L-VOD AGC change represent ± 1 SD of the different L-VOD indices and the ESA CCI biomass map used for calibration.

	Modelled net changes 2012-2019 [Tg C]												[% Tg C]		[Tg C]		[% Tg C]	
Country	Deforestation loss		Edge loss		Degradation loss (non-edge)		Secondary forest growth		Old-growth forest change		Total modelled change		Total modelled relative change		LVOD AGC change		LVOD AGC relative change	
Bolivia	-18.82	±1.6	-8.11	±0.9	-13.96	±1.6	6.44	±0.4	23.32	±16.9	-11.13	±17.1	-0.59	±0.9	-17.63	±12.9	-0.94	±0.7
Brazil	-804.65	±84.4	-290.06	±36.1	-194.00	±30.2	219.69	±14.1	-221.02	±126.5	-1290.04	±159.8	-3.61	±0.4	-1307.37	±411.2	-3.66	±1.2
Colombia	-46.17	±4.1	-16.61	±1.9	-16.19	±2.2	22.71	±1.5	17.20	±33.3	-39.07	±33.7	-0.84	±0.7	-1.44	±24.4	-0.03	±0.5
Ecuador	-13.55	±1.4	-6.92	±0.9	-3.32	±0.4	7.92	±0.5	8.48	±4.0	-7.38	±4.4	-0.88	±0.5	-0.21	±1.4	-0.02	±0.2
French Guiana	-0.86	±0.1	-1.06	±0.1	-1.03	±0.1	0.26	±0.0	-9.73	±3.4	-12.42	±3.4	-1.38	±0.4	-11.47	±3.8	-1.27	±0.4
Guyana	-4.47	±0.4	-4.89	±0.6	-5.64	±0.7	6.96	±0.4	-6.32	±6.0	-14.36	±6.1	-0.73	±0.3	-9.46	±8.0	-0.48	±0.4
Suriname	-4.17	±0.4	-3.34	±0.4	-3.84	±0.5	2.59	±0.2	-9.95	±7.1	-18.71	±7.1	-1.30	±0.5	-12.25	±5.2	-0.85	±0.4
Peru	-70.96	±6.9	-40.83	±4.8	-19.45	±2.3	26.51	±1.7	57.38	±15.6	-47.36	±18.0	-0.83	±0.3	0.21	±3.3	0.00	±0.1
Venezuela	-9.97	±0.8	-11.12	±1.4	-13.14	±1.9	5.32	±0.3	28.15	±23.3	-0.75	±23.4	-0.03	±0.8	37.63	±31.7	1.32	±1.1
Amazon	-973.64	±98.5	-382.97	±46.6	-270.59	±38.6	298.42	±19.2	-112.59	±176.9	-1441.37	±212.1	-2.58	±0.4	-1322.19	±410.2	-2.37	±0.7

Tab. S5: Trends in AGC for Amazon countries from 2011 to 2019 as modelled based on land-cover data and inferred from L-VOD annual AGC respectively. Square brackets represent the 95% confidence interval. Asterisks (*) denote significant trends ($p<0.05$).

				Modelled trends 2011-2019 [Tg C yr^{-1}]			LVOD AGC trends 2011-2019 [Tg C yr^{-1}]		
Country	N cells	AGC 2011		trend		CI	trend		CI
Bolivia	268	1 869.56	±568.6	-2.08		[-4.7, 1.3]	-0.89		[-6.3, 7.1]
Brazil	4 717	35 163.42	±10 705.2	-157.10	*	[-195.4, -104.9]	-142.11	*	[-196.8, -97.2]
Colombia	586	4 659.27	±1 418.4	-4.24		[-14.3, 6.7]	-0.77		[-6.8, 8.5]
Ecuador	92	836.86	±254.7	-1.04	*	[-2.3, -0.3]	-0.28		[-1.7, 1.1]
French Guiana	95	888.09	±270.6	-0.30		[-1.8, 0.3]	-0.13		[-1.7, 0.3]
Guyana	229	1 947.29	±593.5	-0.99		[-2.6, 0.9]	-0.64		[-2.7, 1.3]
Suriname	157	1 431.40	±436.2	-1.88		[-3.8, 0.6]	-0.57		[-3.2, 1.3]
Peru	626	5 697.31	±1 734.8	-7.72	*	[-10.9, -4.5]	-0.49		[-5.2, 5.3]
Venezuela	391	2 836.45	±865.7	1.98		[-6.2, 7.7]	7.02		[-5.1, 13.6]
Amazon	7 162	55 333.55	±16 847.9	-175.92	*	[-220.9, -115.8]	-143.63	*	[-194.5, -85.0]

Tab. S6: Spatial R^2, residual squared error (RSE), Pearson's r and mean absolute error (MAE) between modelled AGC change trends including different processes (All: old-growth forest changes + degradation + deforestation + secondary forest growth) and the L-VOD AGC trends for 0.25° grid-cells, excluding >90% old-growth forest covered cells used as reference for old-growth forest changes.

	All	degradation + deforestation + SF	degradation + deforestation	non-edge degr. + deforestation	deforestation
**R^2**	0.462	0.399	0.402	0.405	0.390
**RSE**	0.544	0.575	0.574	0.573	0.580
**Pearson's r**	0.680	0.632	0.634	0.636	0.624
**MAE**	0.424	0.454	0.485	0.435	0.434

Table S7: Net changes in AGC for Amazon countries from 2011 to 2019 as total change in AGC from L-VOD. Uncertainties represent ± 1 SD of the different L-VOD indices and the ESA CCI biomass map used for calibration.

Country	N cells	AGC 2011		LVOD AGC change		LVOD AGC relative change	
Bolivia	268	1 869.56	±568.6	-6.20	±11.2	-0.33	±0.6
Brazil	4 717	35 163.42	±10 705.2	-777.49	±236.9	-2.21	±0.7
Colombia	586	4 659.27	±1 418.4	-5.06	±15.6	-0.11	±0.3
Ecuador	92	836.86	±254.7	-1.88	±0.9	-0.22	±0.1
French Guiana	95	888.09	±270.6	0.41	±1.5	0.05	±0.2
Guyana	229	1 947.29	±593.5	3.80	±3.9	0.20	±0.2
Suriname	157	1 431.40	±436.2	-2.63	±1.9	-0.18	±0.1
Peru	626	5 697.31	±1 734.8	-9.95	±10.2	-0.17	±0.2
Venezuela	391	2 836.45	±865.7	48.94	±27.8	1.73	±1.0
Amazon	7 162	55 333.55	±16 847.9	-750.18	±228.8	-1.36	±0.4

Tab. S8: Net changes in AGC for Amazon countries from 2011 to 2019 as modelled for each process using land-cover data, ESA CCI biomass map and L-VOD AGC (old-growth forest change), alternative version to Tab. S3 with annual uncertainties combined per process using summation instead of root sum of squares. Uncertainties that arise from the ESA CCI biomass map (± 1 SD, for deforestation, edge and non-edge degradation loss) and from the secondary forest growth model (± 1 SD of average growth rate) are reported. Uncertainties for old-growth forest change represent ± 1 SD of the different L-VOD indices and the ESA CCI biomass map used for calibration. The total modelled change includes the combination of these uncertainties.

				Modelled net changes 2011-2019 [Tg C]												[% Tg C]	
Country	N cells	AGC 2011		Deforestation loss		Edge loss		Degradation loss (non-edge)		Secondary forest growth		Old-growth forest change		Total modelled change		Total modelled relative change	
Bolivia	268	1 869.56	±568.6	-22.93	±4.9	-9.93	±2.9	-19.33	±5.6	7.45	±1.3	17.85	±46.8	-26.89	±47.5	-1.44	±2.5
Brazil	4 717	35 163.42	±10 705.2	-889.48	±232.1	-325.65	±101.5	-210.95	±65.9	247.34	±42.1	123.90	±410.5	-1 054.84	±488.7	-3.00	±1.4
Colombia	586	4 659.27	±1 418.4	-51.94	±11.5	-19.00	±5.7	-17.44	±5.3	25.81	±4.4	36.10	±84.8	-26.47	±86.0	-0.57	±1.8
Ecuador	92	836.86	±254.7	-15.88	±4.0	-7.87	±2.6	-3.57	±1.2	8.95	±1.5	10.23	±11.1	-8.15	±12.2	-0.97	±1.5
French Guiana	95	888.09	±270.6	-0.93	±0.3	-1.18	±0.4	-1.09	±0.3	0.30	±0.1	2.43	±11.5	-0.46	±11.5	-0.05	±1.3
Guyana	229	1 947.29	±593.5	-5.09	±1.2	-5.58	±1.7	-5.97	±1.8	8.06	±1.4	10.89	±20.9	2.30	±21.1	0.12	±1.1
Suriname	157	1 431.40	±436.2	-4.64	±1.2	-3.80	±1.2	-4.05	±1.2	3.00	±0.5	-0.84	±19.6	-10.33	±19.7	-0.72	±1.4
Peru	626	5 697.31	±1 734.8	-79.55	±20.5	-45.75	±14.1	-21.20	±6.6	29.99	±5.1	46.23	±42.9	-70.29	±50.3	-1.23	±0.9
Venezuela	391	2 836.45	±865.7	-11.53	±2.5	-12.22	±3.6	-13.81	±4.1	6.05	±1.0	38.67	±53.1	7.17	±53.5	0.25	±1.9
Amazon	7 162	55 333.55	±16 847.9	-1 081.99	±278.2	-431.01	±133.6	-297.41	±92.0	336.96	±57.3	285.50	±561.2	-1 187.94	±649.5	-2.15	±1.2

Table S9: Net changes in AGC for Amazon countries from 2012 to 2019 as modelled for each process using land-cover data and the total change in AGC from L-VOD, alternative version to Tab. S4 with annual uncertainties combined per process using summation instead of root sum of squares. Uncertainties that arise from the ESA CCI biomass map (± 1 SD, for deforestation, edge and non-edge degradation loss) and from the secondary forest growth model (± 1 SD of average growth rate) are reported. Uncertainties for old-growth forest change represent ± 1 SD of the different L-VOD indices and the ESA CCI biomass map used for calibration. The total modelled change includes the combination of these uncertainties. Uncertainties for the L-VOD AGC change represent ± 1 SD of the different L-VOD indices and the ESA CCI biomass map used for calibration.

	Modelled net changes 2012-2019 [Tg C]												[% Tg C]		[Tg C]		[% Tg C]	
Country	Deforestation loss		Edge loss		Degradation loss (non-edge)		Secondary forest growth		Old-growth forest change		Total modelled change		Total modelled relative change		LVOD AGC change		LVOD AGC relative change	
Bolivia	-18.82	±4.0	-8.11	±2.3	-13.96	±4.1	6.44	±1.1	23.32	±38.6	-11.13	±39.1	-0.59	±2.1	-17.63	±12.9	-0.94	±0.7
Brazil	-804.65	±209.3	-290.06	±90.5	-194.00	±60.8	219.69	±37.4	-221.02	±298.7	-1290.04	±382.5	-3.61	±1.1	-1307.37	±411.2	-3.66	±1.2
Colombia	-46.17	±10.1	-16.61	±5.0	-16.19	±4.9	22.71	±3.9	17.20	±78.0	-39.07	±79.1	-0.84	±1.7	-1.44	±24.4	-0.03	±0.5
Ecuador	-13.55	±3.4	-6.92	±2.2	-3.32	±1.1	7.92	±1.3	8.48	±9.8	-7.38	±10.7	-0.88	±1.3	-0.21	±1.4	-0.02	±0.2
French Guiana	-0.86	±0.2	-1.06	±0.3	-1.03	±0.3	0.26	±0.0	-9.73	±7.6	-12.42	±7.7	-1.38	±0.9	-11.47	±3.8	-1.27	±0.4
Guyana	-4.47	±1.1	-4.89	±1.5	-5.64	±1.7	6.96	±1.2	-6.32	±15.2	-14.36	±15.4	-0.73	±0.8	-9.46	±8.0	-0.48	±0.4
Suriname	-4.17	±1.1	-3.34	±1.0	-3.84	±1.2	2.59	±0.4	-9.95	±16.1	-18.71	±16.3	-1.30	±1.1	-12.25	±5.2	-0.85	±0.4
Peru	-70.96	±18.3	-40.83	±12.6	-19.45	±6.0	26.51	±4.5	57.38	±36.1	-47.36	±43.0	-0.83	±0.8	0.21	±3.3	0.00	±0.1
Venezuela	-9.97	±2.2	-11.12	±3.3	-13.14	±3.9	5.32	±0.9	28.15	±47.9	-0.75	±48.3	-0.03	±1.7	37.63	±31.7	1.32	±1.1
Amazon	-973.64	±249.6	-382.97	±118.8	-270.59	±83.9	298.42	±50.8	-112.59	±439.5	-1441.37	±528.4	-2.58	±0.9	-1322.19	±410.2	-2.37	±0.7
